# Expression and function of the miR-143/145 cluster *in vitro* and *in vivo* in human breast cancer

**DOI:** 10.1371/journal.pone.0186658

**Published:** 2017-10-26

**Authors:** Charles Johannessen, Line Moi, Yury Kiselev, Mona Irene Pedersen, Stig Manfred Dalen, Tonje Braaten, Lill-Tove Busund

**Affiliations:** 1 Department of Medical Biology, UiT—The Arctic University of Norway, Tromsø, Norway; 2 Department of Clinical Pathology, University Hospital of North Norway, Tromsø, Norway; 3 Department of Life Sciences and Health, Oslo and Akershus University College of Applied Sciences, Oslo, Norway; 4 Department of Clinical Medicine, UiT—The Arctic University of Norway, Tromsø, Norway; 5 Department of Community Medicine, UiT—The Arctic University of Norway, Tromsø, Norway; University of South Alabama Mitchell Cancer Institute, UNITED STATES

## Abstract

MicroRNAs (miRNAs) are small non-coding RNAs that function as post-transcriptional regulators of gene expression and are dysregulated in cancer. Studies of miRNAs to explore their potential as diagnostic and prognostic markers are of great scientific interest. Here, we investigate the functional properties and expression of the miR-143/145 cluster in breast cancer (BC) *in vitro* and *in vivo*. The ER positive MCF7, the HER2 positive SK-BR-3, and the triple negative cell line MDA-MB-231 were used to assess cell proliferation and cell invasion. Expression of miRNA in 108 breast cancers in the Norwegian Women and Cancer Study and 44 benign tissue controls were analyzed by microarray and validated by RT-PCR. Further, *in situ* hybridization (ISH) was used to study the cellular and subcellular distribution of the miRNAs. *In vitro*, miR-143 promoted proliferation of MCF7 and MDA-MB-231 cells, whereas miR-145 and the cotransfection of both miRNAs inhibited proliferation in all three cell lines. The cells’ invasive capacity was reduced after transfection and cotransfection of the miRNAs. In line with the tumor suppressive functions *in vitro*, the expression of miR-143 and miR-145 was lower in malignant compared to benign breast tissue, and lower in the more aggressive tumors with higher tumor grade, loss of ER and the basal-like phenotype. ISH revealed miR-143 to be cytoplasmatic and predominantly expressed in luminal cells in benign tissue, whilst miR-145 was nuclear and with strong staining in myoepithelial cells. Both miRNAs were present in malignant epithelial cells and stromal fibroblasts in BC. This study demonstrates that miR-143 and -145 have functional properties and expression patterns typical for tumor suppressors, but the function is influenced by cellular factors such as cell type and miRNA cotransfection. Further, the nuclear functions of miR-145 should be explored for a more complete understanding of the complexity of miRNA regulation and function in BC.

## Introduction

Breast cancer (BC) is the most common cancer diagnosed in women [1]. Clinical classification of BC is done by assessing histological type, grade, stage and receptor status where tumors can be categorized as estrogen receptor positive (ER+), and/or human epidermal growth factor receptor 2 positive (HER2+), or triple negative (TN) [2]. The ER+ BCs constitute nearly 70% of all cases [3], and this group has extended therapeutic options, compared to the HER2+ and TN BCs.

Based on gene expression profiles, the ER+ tumors can be divided into the molecular subtypes luminal A and luminal B, where the luminal B subtype has a worse prognosis due to a higher proliferation rate and/or HER2 positivity [4, 5]. The HER2+ BCs are commonly associated with ductal carcinoma *in situ* (DCIS) and in general have a moderate to poor prognosis [4, 5]. The TN BCs constitute a heterogeneous group of tumors, where the majority of cases are presented as basal-like BCs [6]. The TN BCs typically include the most aggressive breast carcinomas, where the majority of cancer related death occur within five years from time of diagnosis [5, 6]. Molecular profiles are used to guide treatment. However, when comparing individual cases, BCs have highly heterogeneous gene expression contributing to the challenges of treating BC patients [7]. Further, BC is still one of the leading causes of cancer deaths in women [1], underlining the need for improved prognostic and predictive biomarkers for early detection, identification and stratification of the most aggressive tumors, and more targeted treatment.

MicroRNAs (miRNAs) constitute a group of small non-coding endogenous RNAs with a typical length of 18–22 nucleotides. Mature miRNAs bind to the complementary or semi complementary 3’untranslated region (3’-UTR) of mRNAs, resulting in negative regulation of protein translation [8]. The downregulation of protein synthesis can be a result of miRNA induced mRNA degradation, mRNA destabilization, or mRNA silencing [9]. The nature of the negative regulation is dependent upon the degree of complementarity between the mature miRNA and the 3’-UTR target [9]. Due to the highly pleiotropic nature of miRNAs, it is predicted that more than 60% of all human protein coding genes are influenced by miRNAs, and their dysregulation is a universal event for virtually all types of malignancies, as they have a profound influence on most cellular processes [10–12]. Expression profiles of miRNA have been shown to categorize various cancers more accurately than mRNA [13], and miRNAs can be considered novel regulators in the hallmarks of human cancers [14]. Combined with miRNAs’ biochemical properties that make them suitable as biomarkers, it is of great scientific interest to investigate and characterize individual miRNAs, their expression, and their functional roles in BC and BC subtypes.

MiR-143 and miR-145 constitute a miRNA cluster and appear to have tumor suppressor functions in a variety of organ systems, both as individual miRNAs and as a cluster [15–24]. This study evaluates the miR-143 and miR-145 expression profile in an unselected cohort of BC within the Norwegian Women and Cancer Study (NOWAC) postgenome cohort [25]. Samples were stratified in subgroups based on molecular subtype, receptor status, tumor grade and lymph node status. In addition, through a series of *in vitro* experiments, including assays for cell proliferation and cell invasion, the functionality of miR-143 and miR-145 was studied in BC cell lines analogous to the major subtypes of breast cancer; ER+, HER2+ and TN BC.

## Materials and methods

### Ethics statement

The study of miRNA expression in BC samples from the NOWAC postgenome cohort and benign breast tissue has been approved by the regional ethical committee of North Norway (REKnord 2010/1931, 2013/2271). The Data Inspectorate has also approved the storing of relevant, not identifiable data and the linkage to national registries. In addition, ethical aspects have been considered within the project to ensure the most efficient and accurate use of the material collected and data generated, in accordance with national and international guidelines and laws.

### Functional studies

The potential function of miR-143 and miR-145 in tumorigenesis was investigated by a series of *in vitro* experiments. The experiments were performed by introducing miR-143 mimic or miR-145 mimic, alone or in combination, alongside a miRNA negative control into various BC cell lines. In this study, cell proliferation and cell invasion were assessed.

#### Cell cultures

The functions of miR-143 and miR-145 were evaluated in three different BC cell lines. These included the ER+ MCF7 (ATCC ® HTB-22™), the HER2+ SK-BR-3 (ATCC® HTB-30™), and the TN BC cell line MDA-MB-231 (ATCC® CRM-HTB-26™). All cell lines, except MCF7, were cultured in RPMI-1640 media (cat.# R8758, Sigma-Aldrich, St. Louis, USA) supplemented with 10% fetal bovine serum (cat.# S0415, Biochrom, Berlin, Germany). MCF7 were cultured in DMEM (cat.# D5796, Sigma-Aldrich, St. Louis, USA) with the same supplements as the previously described cell lines. All cell lines were incubated at 37°C in humidified atmosphere with 5% CO_2_. Total RNA from the non-cancerous breast cell line MCF-10A was a kind gift from the research group of professor E. Mortensen, RNA and molecular pathology (RAMP) research group, UiT—The Arctic University of Norway, Tromsø, Norway.

#### Cell transfection

All cell lines were transiently transfected with 100 nM hsa-miR-143-3p Pre-miR™ miRNA Precursor (cat.# PM10883, Thermo Fisher Scientific, USA) and/or 100 nM hsa-miR-145-5p Pre-miR™ miRNA Precursor (cat.# PM11480, Thermo Fisher Scientific, USA), alongside the Cy3™ Dye-Labeled Pre-miR Negative Control #1 (cat.# AM17120, Thermo Fisher Scientific, USA). The transfection was performed by using 6 μl/mL of the Lipofectamine® RNAiMAX transfection reagent (cat.# 13778075, Thermo Fisher Scientific, USA). Transfected Cy3™ Dye-Labeled Pre-miR Negative Control emits fluorescent light when exposed to UV-light, and the transfection efficiency was determined using a fluorescence microscope. The transfection efficiency was typically as high as 80–95%.

#### Total RNA isolation

Total RNA was isolated from cell lines using the miRNeasy Mini Kit (cat.# 217004, Qiagen, Hilden, Germany) according to the manufacturer’s protocol. In short, cells were lysed in 700 μl QIAzol Lysis Reagent before homogenization and 5 minutes incubation at room temperature. 140 μl chloroform were added, and the samples were shaken before incubation at room temperature for 3 minutes. Samples were centrifuged for 15 minutes at 12000 g at 4°C, and the upper aqueous phase was transferred and mixed thoroughly with 100% ethanol. The samples were transferred into the RNeasy® Mini column and washed in several steps before elution with 50 μl ddH_2_O. Isolated total RNA samples were stored at -70°C.

#### cDNA synthesis

First strand cDNA synthesis was performed using the miScript II RT Kit (cat.# 218160, Qiagen, Hilden, Germany) according to the manufacturer’s protocol. Briefly, 100 ng of total RNA was mixed with 4 μl 5x miScript HiSpec Buffer, 2 μl 10x Nucleics Mix, 2 μl miScript Reverse Transcriptase Mix, and RNase-free water to a total volume of 20 μl. Samples were incubated for 60 minutes at 37°C, and subsequently incubated for 5 minutes at 95°C to inactivate enzymes. Finally, samples were diluted up to a total volume of 200 μl in RNase-free water and stored at -20°C.

#### RT-PCR

Endogenous levels of miR-143 and miR-145 in the selected cell lines were quantified relative to the stably expressed reference snRNA RNU6 using real-time PCR and the miScript SYBR® Green PCR Kit (cat.# 218073, Qiagen, Hilden, Germany). Primers used were miScript Primer Assays Hs_miR-143_1 miScript Primer Assay (cat.# MS00003514, Qiagen, Hilden, Germany), Hs_miR-145_1 miScript Primer Assay (cat.# MS00003528, Qiagen, Hilden, Germany) and Hs_RNU6-2_11 miScript Primer Assay (cat.# MS00033740, Qiagen, Hilden, Germany), according to the manufacturer’s protocol. Briefly, a total volume of 25 μl/well in a 96-well plate included 1 μl cDNA mixed with 12.5 μl 2x QuantiTect SYBR Green PCR Master Mix, 2.5 μl 10x miScript Universal Primer, 2.5 μl 10x miScript Primer Assay, and 6.5 μl RNase-free Water. The plate was sealed and centrifuged for 1 minute at 1000 g before it was placed in a 7300 Real-Time PCR System (Thermo Fisher Scientific, Waltham, Massachusetts, USA). Each sample was analyzed in quadruplicates, and three independent experiments were performed.

#### Proliferation assay

The BC cell lines’ ability to proliferate after transfection was evaluated using the real-time cell analyzer system xCelligence, RTCA DP (cat#05469759001, ACEA Biosciences, San Diego, USA) fitted with the E-plate 16 (cat#05469830001, ACEA Biosciences, San Diego, USA). Prior to analysis on the xCelligence platform, cell lines were trypsinized until detached, resuspended in complete growth media, and counted. Initial titration experiments estimated approximately 8000 cells per well to be optimal. In accordance with the manufacturer’s protocol, cells were seeded in quadruplicates into an E-plate after baseline measurements. The E-plate containing cells was incubated for 30 minutes at room temperature before positioned in the RTCA DP instrument, which was located in an incubator preserving the same conditions as used for routine cultivation of cell lines. The instrument denotes the cellular growth rate as ‘Cell Index’, which is an arbitrary unit reflecting the cell-sensor impedance. The cell index was recorded by the instrument every 30 minutes. Growth curves were calculated with the RTCA software version 1.2.1 (ACEA Biosciences, San Diego, USA). A minimum of three independent experiments were performed for each cell line.

#### Invasion assay

The cell lines invasiveness after transfection was tested using the CytoSelect^TM^ 96-well Cell Invasion Assay, Basement membrane (cat.# CBA-112, Cell Biolabs, San Diego, USA) according to the manufacturer’s protocol. Briefly, 50000 pretransfected and serum starved cells were seeded in the upper chamber of a modified Boyden chamber. The chamber was coated with a basement membrane consisting of a protein matrix isolated from Engelbreth-Holm-Swarm tumor cells, and the cells were allowed to invade for 24 h towards the bottom chamber containing media+10% FBS. Cancer cells able to invade the basement membrane and pass through the porous membrane to the bottom side of the membrane were lysed, stained, and fluorescence was measured at 480/520 nm using the CLARIOstar® microplate reader (BMG LABTECH, Ortenberg, Germany). All experiments were performed in quadruplicates, and a minimum of three independent experiments were performed for each cell line.

### Patient material and tumor classification

The patient samples were collected from the NOWAC postgenome cohort [25]. The NOWAC participants included in this study were diagnosed with breast cancer at the Department of Pathology at the University Hospital of North Norway in Tromsø, or the Nordland Hospital in Bodø in the years 2004–2010. Archived formalin-fixed paraffin-embedded (FFPE) tissue blocks, and hematoxylin and eosin stained slides were collected. Histological grading of tumors was based on the criteria modified by Elston and Ellis [26] and immunohistochemical (IHC) analyses of ER, progesterone receptor (PR) and HER2 were done on needle biopsies as part of routine diagnostics. The cut-off value for ER positivity was ≥ 1%, for PR ≥ 10% and a HER2 score of 3+ was considered positive, a score of 0–1+ negative whereas a score of 2+ lead to silver in situ hybridization (SISH) where HER2 was considered negative if HER2/chromosome 17-ratio was < 2. IHC staining for the proliferation marker Ki67 was done on slides from the primary surgery, and the expression evaluated in at least 500 tumor cells in the most proliferative areas of the tumor and the result reported as a percentage of positive tumor cells. Subtyping of the tumors according to molecular profile were based on the surrogate markers ER, PR, HER2 and Ki67 according to recommendations by the St Gallen International Expert Consensus and previous publications [27, 28]. The subtyping was performed as follows: luminal A (ER+ and/or PR+, HER2- and Ki67 ≤ 30%), luminal B (ER+ and/or PR+, HER2- and Ki67 > 30% or ER+ and/or PR+ and HER2+), HER2 positive (ER- and PR- and HER2+) and basal-like (ER-, PR- and HER2-). Histopathological data were collected from the original pathology reports, and reevaluated and completed according to updated criteria by a breast pathologist (L.M.). As benign tissue controls, FFPE tissue cores from 44 breast reduction surgery specimens were included in the study.

### miRNA microarray

Total RNA was extracted from FFPE tissue cores from both malignant and benign breast tissue using the RecoverAll Total Nucleic Acid Isolation kit (Life Technologies, Grand Island, NY, USA) following the manufacturer’s instructions. RNA quality and quantity was assessed using the NanoDrop 1000 spectrophotometer (Thermo Fisher Scientific, Wilmington, DE). Microarray hybridization and analyses were performed as a bought service by Exiqon (Vedbaek, Denmark). In short, the miRCURY LNATM microRNA Hi-Power Labeling Kit (Exiqon) was used to label 250 ng total RNA from samples and reference with Hy3TM and Hy5TM, respectively. The Hy5TM -labeled reference RNA contained an equal aliquot of all RNA species included in the study. Labeled samples and reference RNA were mixed before hybridization to the 7th generation miRCURY LNA microRNA array (Exiqon), using a Tecan HS4800 hybridization station (Tecan, Austria). The microarray contained capture probes for miRNAs in human, mouse and rat as annotated in miRBASE version 19.0. The slides were scanned on the Agilent G2565BA Microarray Scanner System (Agilent technologies Inc., USA) and the ImaGene 9.0 software (BioDiscovery Inc., USA) was used for image analysis. The quantified signals were background corrected and normalized using quantile normalization method and detection threshold set as 1.2 times the 25th percentile of the overall signal intensity of the individual slides.

### Validation of microarray and quantification of miRNAs by RT-qPCR

Microarray miRNA analyses were validated using RT-qPCR. 40 tumor samples representing the four major molecular subtypes of cancer included in the study, and 20 of the benign breast tissue controls were included in the PCR validation done by Exiqon. In short, RNA was extracted from FFPE tissue cores using the Qiagen miRNeasy FFPE kit according to the manufacturer’s instructions (Qiagen, Hilden, Germany). 10 ng RNA was reverse transcriped using the miRCURY LNA Universal RT microRNA PCR, Polyadenylation and cDNA synthesis kit (Exiqon) and PCR-reactions performed on 100 x diluted cDNA using ExiLENT SYBR Green master mix. The amplification was done in a Light Cycler 480 Real-Time PCR System (Roche) in 384 well plates. All reverse transcription reactions were done in duplicates. Based on stable expression across the data set, the most suitable reference miRNAs were evaluated by Exiqon using the Normfinder software. Of the suitable reference miRNAs, miR-664a-3p was detected in all samples and was used for normalization. Normalized expression values for each miRNA in each sample were calculated using the quantification cycle (Cq) from PCR analyses and the formula: average Cq (all samples)–assay Cq (sample).

### *In situ* hybridization

In order to study the cellular and subcellular location of miR-143 and miR-145 in benign and malignant breast tissue, we analyzed the miRNA *in situ* hybridization (ISH) staining in full histological slides of 16 tumors with adjacent normal tissue. Buffers and detection reagents were purchased from Roche (Basel, Switzerland) and labelled locked nucleic acid (LNA) modified probes purchased from Exiqon (Vedbaek, Denmark). The chromogen ISH was performed in the Ventana Discovery Ultra instrument for IHC and ISH (Ventana Medical Systems Inc, Arizona, USA) with deparaffinization, pretreatment, hybridization, chromogen staining and counterstaining automatized in the instrument. In short, 4 **μ**m tissue sections were incubated overnight at 60°C to attach tissue to Super Frost Plus slides. To ensure good distribution of reagents and protect sections from drying, liquid coverslip oil (Roche) was added during incubation. Sections were deparaffinized in EZ Prep buffer (Roche) at 68°C (3 x 12 min), followed by heat-mediated retrieval pretreatment at 95°C with CC1 buffer (Roche) for 40 minutes and rinsing with Reaction Buffer (Roche) followed by RiboWash SSPE buffer (Roche). In this study, we used 5 nM miR-145-5p target probe, 10 nM miR-143-3p target probe, 10 nM scramble miR negative control probe and 0.5 nM U6 positive control probe. Positive and negative tissue controls for both miRNAs were included by using a TMA multi-organ slide.

All slides were denaturated for 8 min at 90°C, hybridization with probes took place for 60 min at 50°C for miR-145, 55°C for miR-143, 57°C for scramble miR and 55°C for U6. Stringent washes were done 2 x 8 minutes with 2.0X RiboWash SSPE, followed by rinsing with Reaction Buffer and blocking against unspecific binding with blocking solution (Roche) for 16 minutes at 37°C. Immunological detection was done with prediluted alkaline phosphatase (AP)-conjugated anti-DIG (Roche) at 37°C for 20 minutes. The sections were rinsed with Reaction Buffer and EZ Prep before the substrate enzymatic reactions were carried out with NBT/BCIP (CromoMap Blue kit, Roche) for 60 minutes at 37°C. Sections were rinsed with Reaction Buffer and counterstained for 4 minutes with Red Stain II (Roche). Dehydration of the sections was performed by increasing gradients of ethanol and finally the tissue sections were mounted with glass cover slips.

#### Scoring of ISH staining intensity

ISH stained full slides of selected tumors were used to collect information on staining intensity and density in tumor cells, stromal fibroblast and adjacent normal breast tissue. The tumors were randomly selected from histological slides of good quality with well preserved invasive carcinoma present. For each slide, three areas of tumor tissue and tumor-associated stromal tissue were evaluated using a microscope at 200x magnification. By morphologic criteria, the tumor cells and stromal fibroblasts were scored for staining intensity with the dominant staining intensity scored as: 0 = negative, 1 = weak, 2 = moderate, 3 = strong. From the observed ISH staining pattern, both tumor cells and stromal fibroblasts stained diffusely and homogenously and hence staining density was not scored since it did not give any additional information. All samples were independently scored by two experienced pathologists (L.M. and S.M.D.).

### Statistics

The miRNA microarray and PCR expression data from the breast cancer samples in the NOWAC study were analyzed using the Limma package in R (Linear Models for Microarray and RNA-Seq Data). Moderated F-statistics were applied, with p-values corrected for multiple testing by controlling the false discovery rate using the method of Benjamini & Hochberg. Descriptive statistics, non-parametric tests and correlation analysis were performed using Stata, version 14.

For the qPCR results from the *in vitro* experiments, the standard error was calculated using all four technical replicates from a representative biological experiment. For the proliferation study, each proliferation curve was tested against the miRNA control using one-way ANOVA with p-values corrected for multiple testing by controlling the false discovery rate using the method of Benjamini & Hochberg. For the invasion study, the standard error was calculated using all four technical replicates from a representative biological experiment.

## Results

### Relative expression of miR-143 and miR-145 in breast cancer

**Fig 1:**
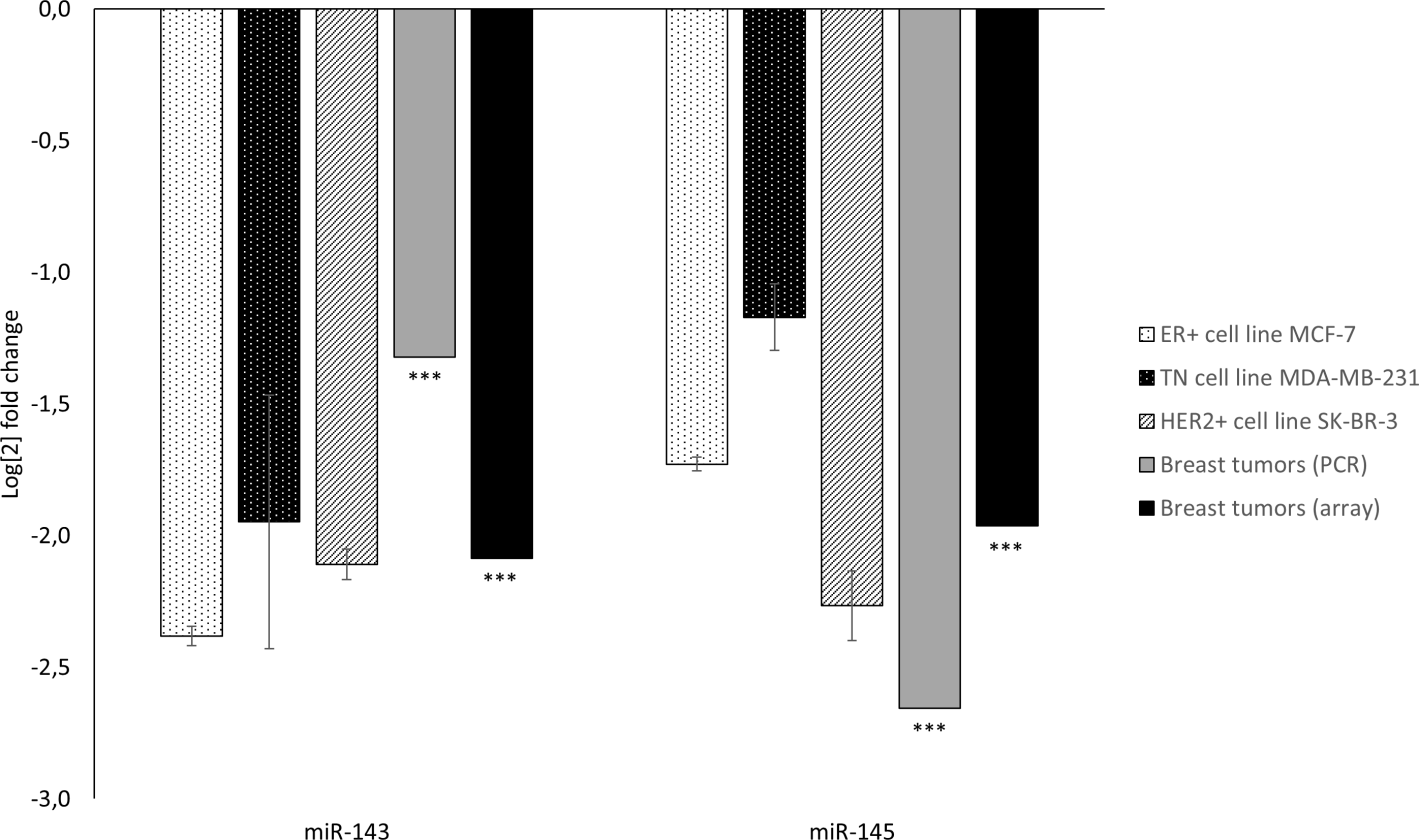
MiRNA expression in breast cancer cell lines and tumor tissue relative to non-cancerous control. Endogenous expression of miR-143 and miR-145 in breast cancer cell lines compared to the non-cancerous breast cell line MCF-10A and in breast cancer tumors compared to benign breast tissue, analyzed using PCR or microarray technology. Data from cell lines are presented as mean log fold change ±SE from a representative biological replicate using all four technical replicates. *** signifies P<0.001 using moderated F-statistics and the Benjamini & Hochberg correction.

The endogenous expression of miR-143 and miR-145 in the studied BC cell lines was quantified relative to the non-cancerous cell line MCF-10A (Fig 1). Relative to MCF-10A, endogenous expression levels of miR-143 and miR-145 were downregulated in all BC cell lines. This pattern was also evident in the NOWAC patient material, as described later.

### Functional studies on miR-143 and miR-145 *in vitro*

The potential functional role of miR-143 and miR-145 in breast cancer tumorigenesis was explored by a series of *in vitro* experiments. Proliferation and invasion were assessed after transfecting BC cell lines with either miR-143, miR-145, or miR-143 and miR-145 in combination.

#### miR-143 promotes proliferation *in vitro*

**Fig 2:**
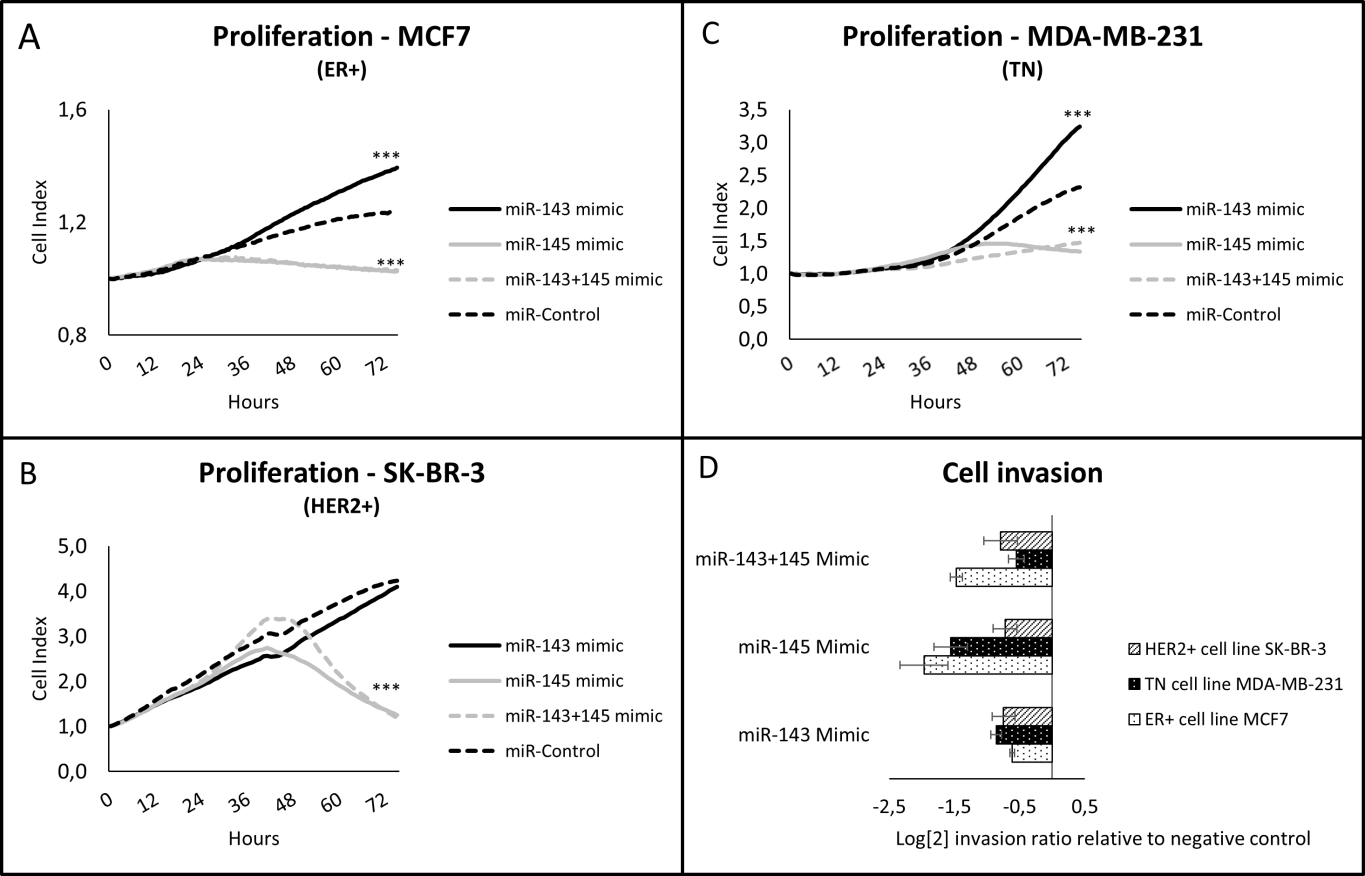
Functional studies on BC cell lines after transfection with miR-143 and/or miR-145. ER+ MCF7 cells (panel A), HER2+ SK-BR-3 cells (panel B) and triple negative MDA-MB-231 cells (panel C) were transfected with miR-143 mimic, miR-145 mimic, either alone or in combination, or with control mimic, and cell proliferation monitored in real time using xCelligence. Data presented are representative for all three biological replicates, and include four technical replicates for each transfection. *** signifies P<0.001 using one-way ANOVA and the Benjamini & Hochberg correction comparing miR-143 and/or miR-145 transfected cells to controls. The invasion capacity (panel D) of all BC cell lines transfected with either miR-143, miR-145, or miR-143 and miR-145, was studied using the CytoSelect Cell Invasion Assay with chambers separated by a basement membrane. Results are presented as mean log fold change ± SE of transfected cells relative to negative controls from a representative biological experiment with four technical replicates for each cell line.

The proliferation rate of the BC cell lines was assessed using the real-time monitoring system xCelligence, fitted with a proliferation plate. Interestingly, transfection of miR-143 led to increased proliferation in the ER+ cell line MCF7 and the TN cell line MDA-MB-231 (Fig 2A and 2C). The HER2+ cell line, however, did not demonstrate any significant change in proliferation when transfected with miR-143 (Fig 2B).

#### miR-145 inhibits proliferation *in vitro*

All three studied BC cell lines demonstrated a dramatic drop in proliferation when transfected with miR-145 (Fig 2A–2C). The shift in proliferation capacity occurred between 24–48 hours after transfection, depending on the cell line.

#### miR-143 and miR-145 inhibits invasion *in vitro*

The cells’ invasive abilities were studied using a Boyden chamber assay. Pretransfected cells were seeded in serum-free media in the upper chambers, and allowed to invade through a basement membrane extract towards the bottom chamber containing media with 10% serum. Invading cells were quantified 20–24 hours after being seeded to the upper chamber. Both miR-143 and miR-145 had a profound effect on cell invasion (Fig 2D). All cell lines demonstrated reduced invasive capacity 24 hours after transfection.

#### Cotransfection of miR-143 and miR-145 results in a tumor suppressor phenotype

In addition to investigating the functional effects of each individual miRNA, we also wanted to study the effects of cotransfecting miR-143 and miR-145. The BC cell lines were simultaneously transfected with 50 nM of both miR-143 and miR-145. The proliferation of cells cotransfected with miR-143 and miR-145 was dramatically reduced in all three cell lines and was similar to the proliferation pattern of cells transfected with miR-145 alone (Fig 2A–2C). The proliferation promoting effects observed for miR-143 in the ER+ cell line and the TN cell line were cancelled by the simultaneous transfection of miR-145 (Fig 2A and 2C). Cotransfection of miR-143 and miR-145 had an inhibitory effect on invasion, in line with the observations made for each individual miRNA (Fig 2D). The cumulative effect of cotransfection using both miRNAs was not significantly different from experiments where only one miRNA was used.

### Patient material

A total of 108 NOWAC postgenome cohort participants were diagnosed with BC at the pathology departments in Northern Norway in the years 2004–2010 and included in the study. Of these, one case had no FFPE tissue block with enough tumor tissue for further analyses. Five cases and six of the benign tissue controls had poor RNA quality, leaving 102 BC surgery specimens and 38 of the 44 benign breast specimens to be included in the miRNA microarray. After microarray miRNA analyses, an additional case and two of the controls were identified as outliers and excluded from further statistical analyses. The histopathological variables for the study cohort are presented in Table 1.

**Table 1:** Histopathological variables for the breast cancer cases included in the study.

NOWAC study		
Variables		N (%)
**Study subjects**	**All**	**108 (100)**
**Tumor size**	≤10 mm	23 (21.3)
	11–20 mm	50 (46.3)
	>20 mm	34 (31.5)
	Unknown	1 (0.9)
**Histological grade**	1	34 (31.5)
	2	42 (38.9)
	3	28 (25.9)
	Unknown	4 (3.7)
**Receptor status**	HR+/HER2-	70 (64.8)
	HR+/HER2+	11 (10.2)
	HR-/HER2+	9 (8.3)
	HR-/HER2-	16 (14.8)
	Unknown	2 (1.9)
**Lymph node met**	No	73 (67.6)
	Yes	34 (31.5)
	Unknown	1 (0.9)
**Molecular subtype**	Luminal A	58 (53.7)
	Luminal B	22 (20.4)
	HER2+	9 (8.3)
	Basal-like	16 (14.8)
	Unknown	3 (2.8)

### Expression of miR-143 and miR-145 in benign and malignant breast tissue and according to histopathological parameters: microarray and PCR-results

**Fig 3:**
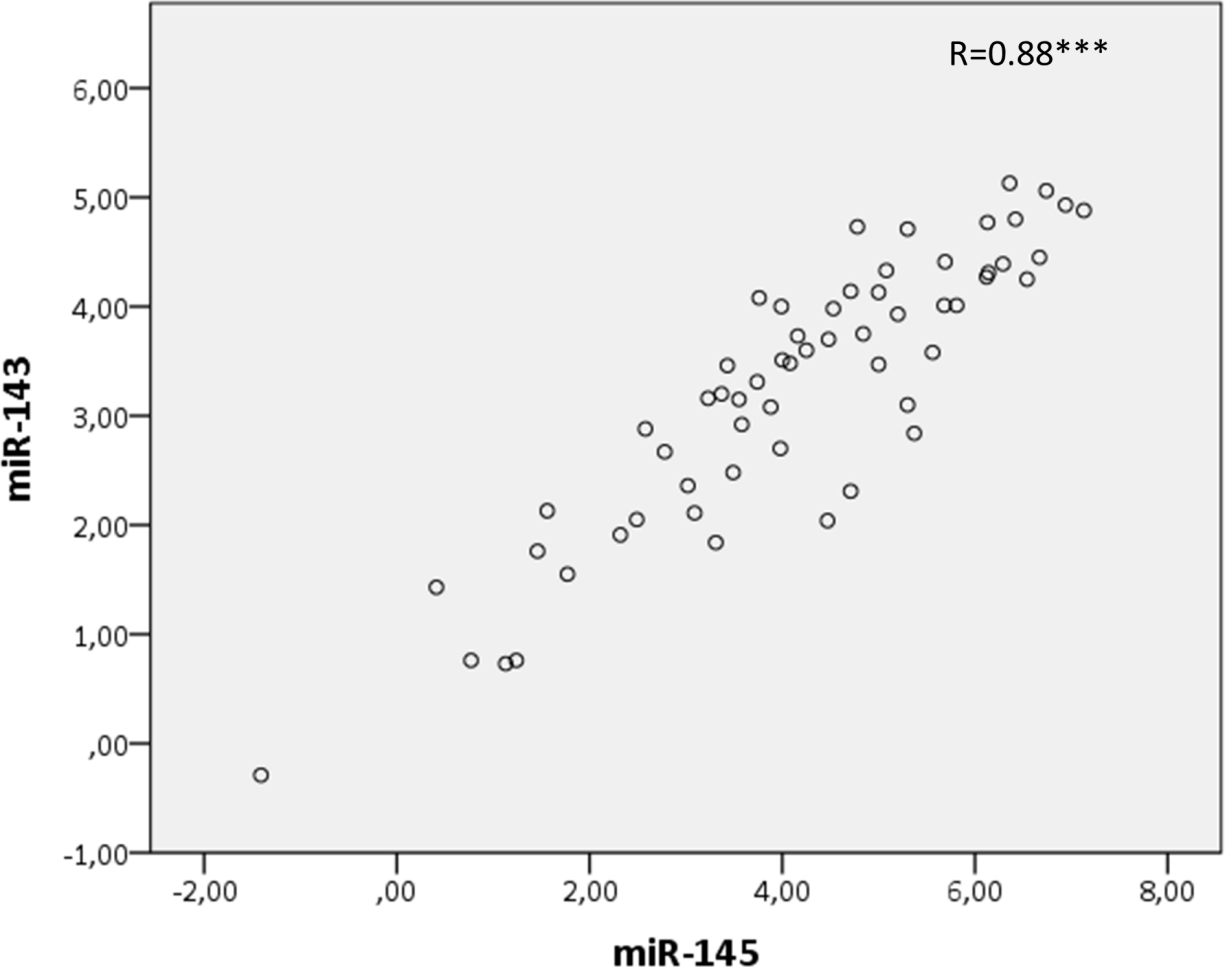
Scatterplot of miR-143 and miR-145 expression in breast tumor tissue. Scatterplot of miR-143 and miR-145 expression in tumor tissue, measured by PCR. Spearman’s rho (R) is presented in the figure (***P<0.001).

Microarray miRNA analyses demonstrated that miR-143 and miR-145 were significantly downregulated in BC tissue compared to benign breast tissue (p<0.001 for both comparisons) and the downregulation was validated and confirmed by PCR (p<0.001) (Fig 1). Of note, microarray- and PCR-based expression levels were significantly correlated for both miR-143 (r = 0.60, p<0.001) and miR-145 (r = 0.72, p<0.001). Additionally, the expression levels of miR-143 and miR-145 were highly correlated (R = 0.88, p<0.001, Fig 3).

**Fig 4:**
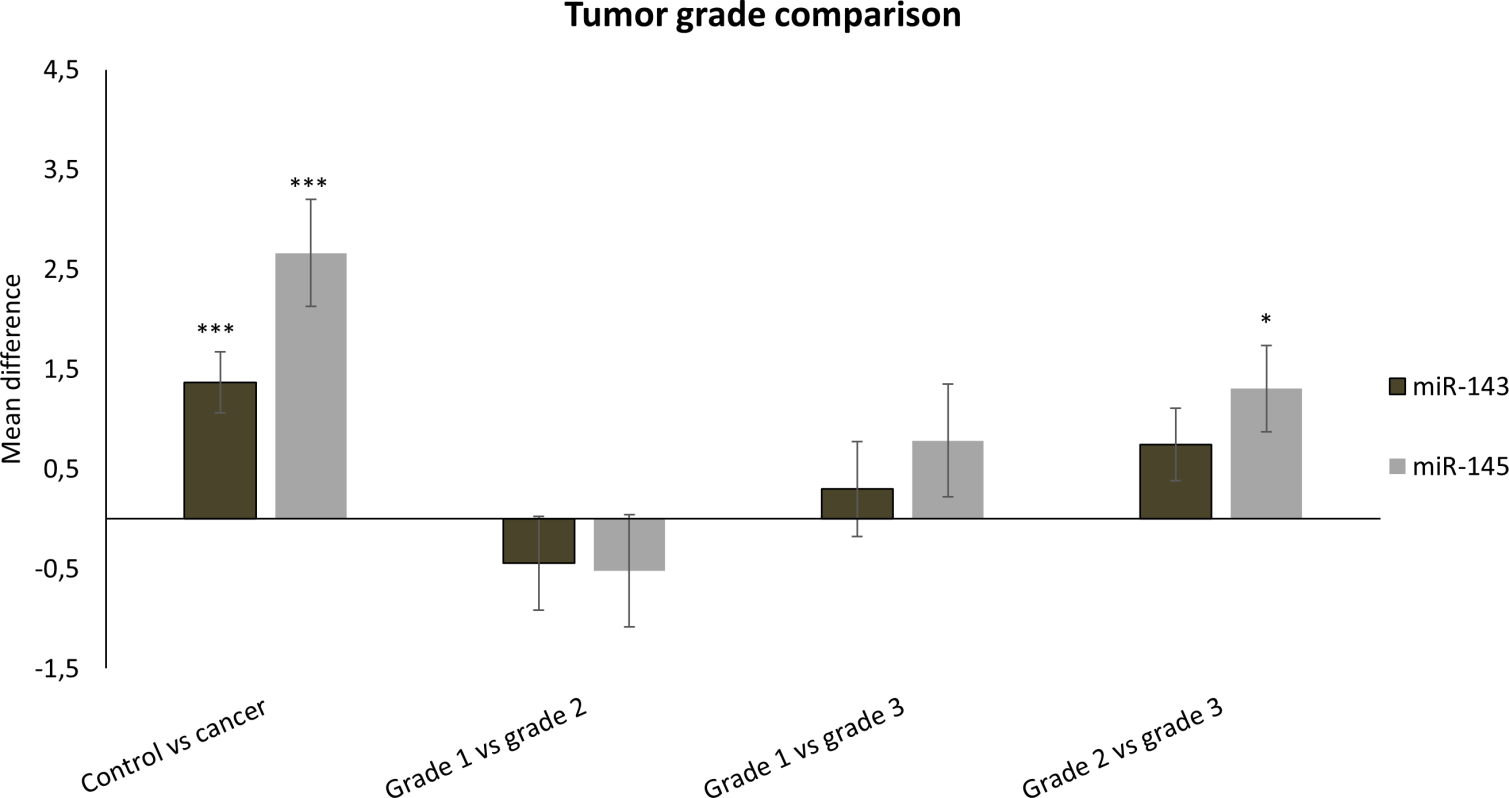
MiRNA expression in tumor tissue, stratified according to histological grade. Mean differences ±SD in miRNA expression in tumor versus benign breast tissue and between tumors stratified according to histological grade, are presented. Statistics are calculated using one-way ANOVA with p-values corrected for multiple testing (*P<0.05, ***P<0.001).

**Fig 5:**
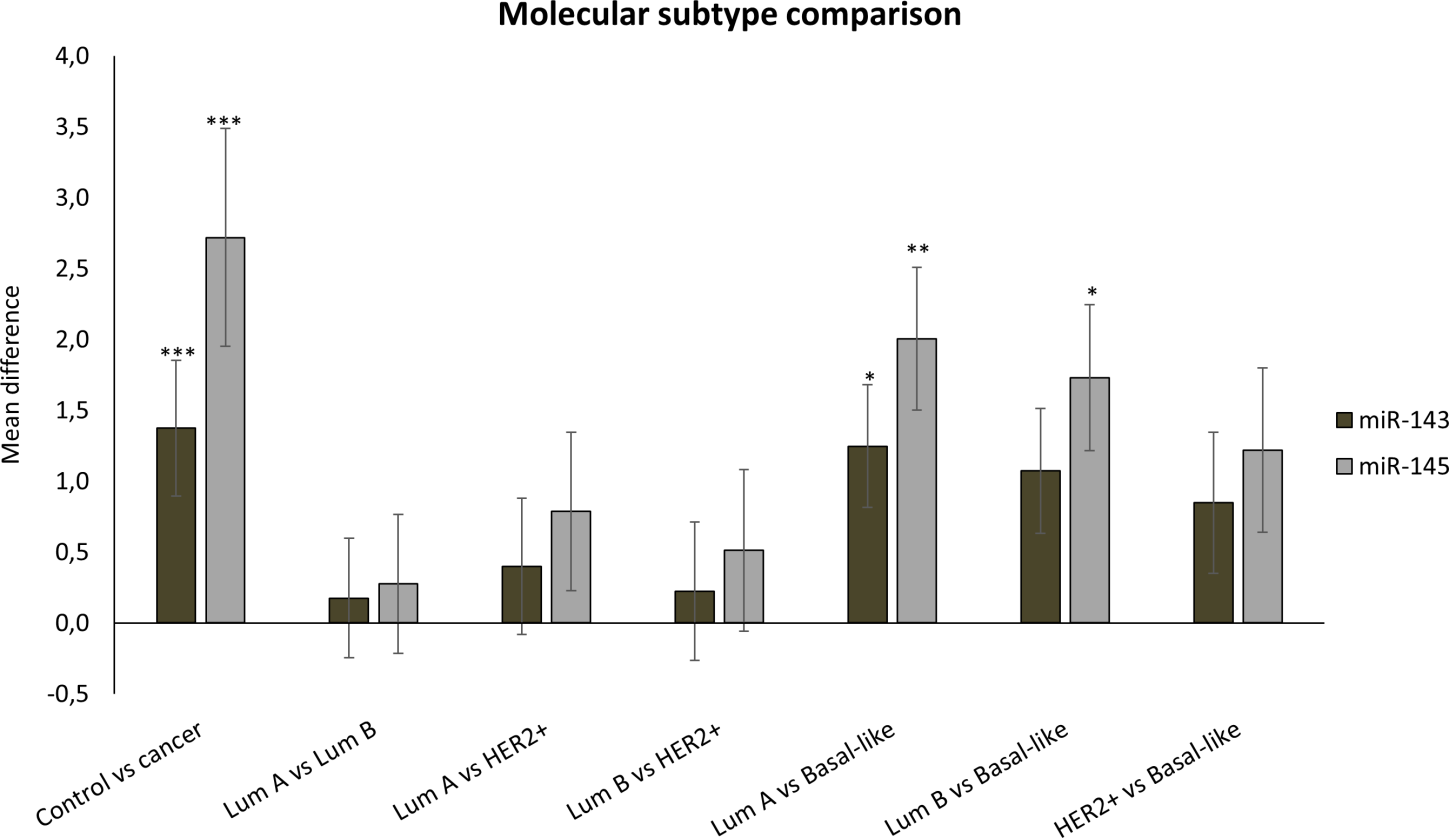
MiRNA expression in tumor tissue, stratified according to molecular subtype. Mean differences ±SD in miRNA expression between tumors stratified according to molecular subtype are presented. Statistics are calculated using one-way ANOVA with p-values corrected for multiple testing. (*P<0.05, **P<0.01, ***P<0.001).

**Fig 6:**
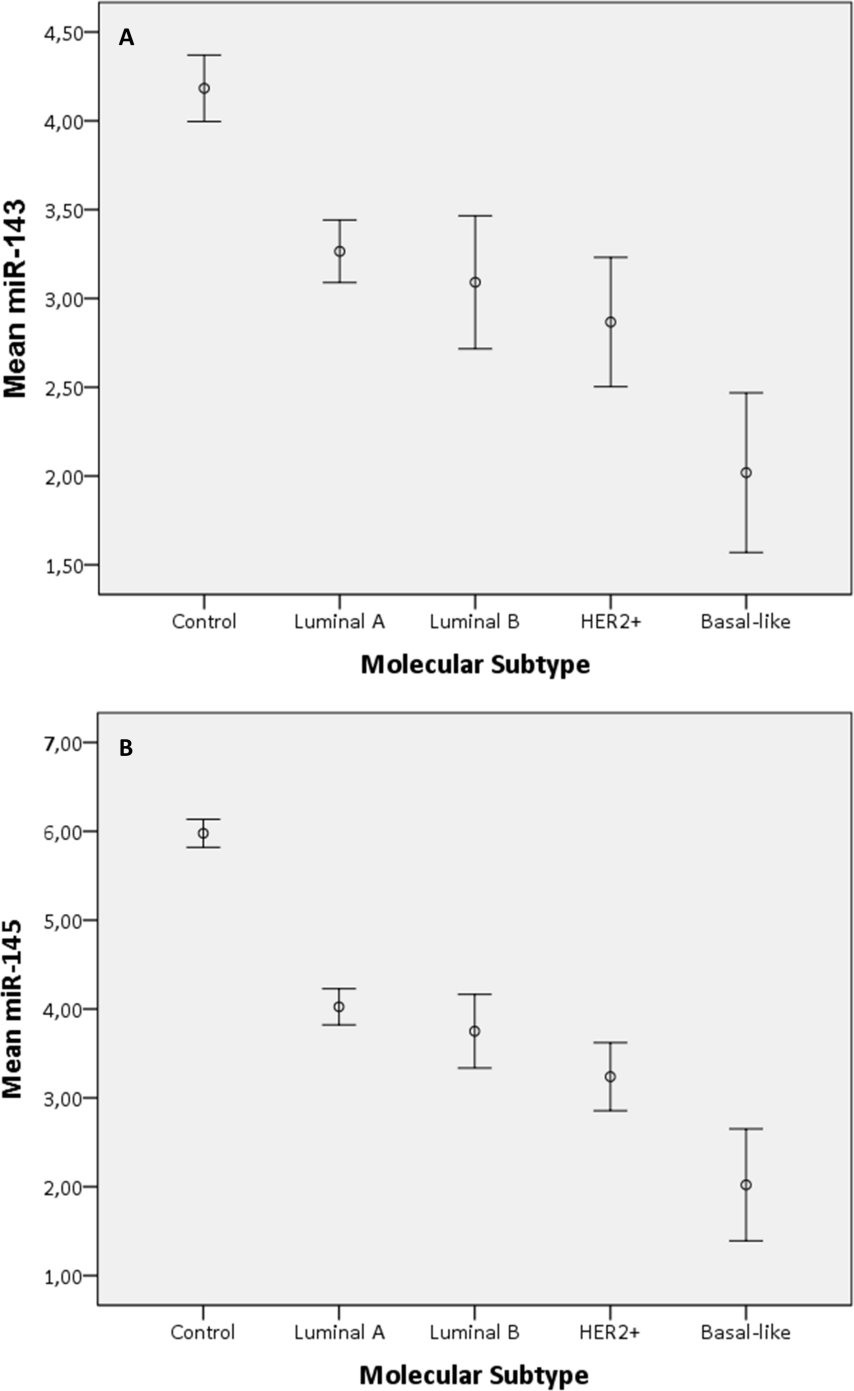
Means plot of miR-143 and miR-145 in breast tumors according to molecular subtype. Means plot for the expression of miR-143 (panel A) and miR-145 (panel B) according to molecular subgroup, using PCR. Error bars describe the standard error in each subgroup.

Based on PCR measurements, both miR-143 and miR-145 demonstrated significantly higher expression in tumors that typically have a better prognosis compared to the rest. There was higher expression of both miR-143 and miR-145 in low and intermediate grade tumors compared to the high grade tumors, as shown in Tables 2 and 3 and Fig 4, and in ER-positive compared to ER-negative tumors (Table 4). MiR-145 displayed higher expression in luminal A tumors compared to the other molecular subtypes, and the expression of both miRNAs was lower in basal-like tumors compared to the other major subtypes (Table 5 and Fig 5). The means plot for miR-143 and miR-145 in Fig 6A and 6B, respectively, illustrates the distribution of expression across molecular subtypes. The same trends were observed in the microarray results, but the differences were not statistically significant. There were no significant differences in miR-143 and miR-145 expression between tumor groups stratified according to tumor size or lymph node metastases.

**Table 2:** Descriptive data for miR-143 and miR-145 PCR measurements in breast tumors according to histological grade.

Tumor grade		miR-143	miR-145
**Control**	**Mean**	4.18	5.98
	**SD**	0.81	0.69
	**N (%)**	19 (100)	19 (100)
**All cancers**	**Mean**	2.82	3.32
	**SD**	1.16	1.53
	**N (%)**	40 (100)	40 (100)
**Tumor grade 1**	**Mean**	2.77	3.40
	**SD**	1.07	1.21
	**N (%)**	7 (17.5)	7 (17.5)
**Tumor grade 2**	**Mean**	3.21	3.92
	**SD**	0.85	1.05
	**N (%)**	17 (42.5)	17 (42.5)
**Tumor grade 3**	**Mean**	2.47	2.62
	**SD**	1.41	1.84
	**N (%)**	16 (40)	16 (40)

**Table 3:** Contrast tests of miR-143 and miR-145 PCR measurements in breast tumors according to histological grade.

miRNA	Test	Mean difference	P*
**miR-143**	Control vs cancer	1.36	**<0.001**
	Grade 1 vs 2 and 3	-0.07	0.88
	Grade 1 and 2 vs 3	0.52	0.17
	Grade 1 vs 3	0.30	0.56
**miR-145**	Control vs cancer	2.66	**<0.001**
	Grade 1 vs 2 and 3	0.13	0.82
	Grade 1 and 2 vs 3	1.04	**0.03**
	Grade 1 vs 3	0.78	0.23

*False discovery rate (FDR) adjusted P-value

**Table 4:** Contrast tests of miR-143 and miR-145 PCR measurements in breast tumors according to ER-status.

miRNA	ER-positive (n = 23)	ER-negative (n = 17)	Mean difference	P*
**miR-143**	3.18	2.37	0.81	**0.02**
**miR-145**	3.89	2.52	1.37	**<0.001**

*FDR adjusted P-value

**Table 5:** Contrast tests of miR-143 and miR-145 PCR measurements in breast tumors according to molecular subtype.

miRNA	Test	Mean difference	P*
**miR-143**	Control vs cancer	1.36	**<0.001**
	Luminal A vs others	0.61	0.13
	Luminal B vs others	0.37	0.36
	HER2+ vs others	0.08	0.88
	Basal-like vs others	-1.06	**0.01**
**miR-145**	Control vs cancer	2.66	**<0.001**
	Luminal A vs others	1.02	**0.02**
	Luminal B vs others	0.66	0.11
	HER2+ vs others	-0.03	0.96
	Basal-like vs others	-1.65	**<0.001**

*FDR adjusted P-value

### ISH expression of miR-143 and miR-145 in benign and malignant breast tissue

**Fig 7:**
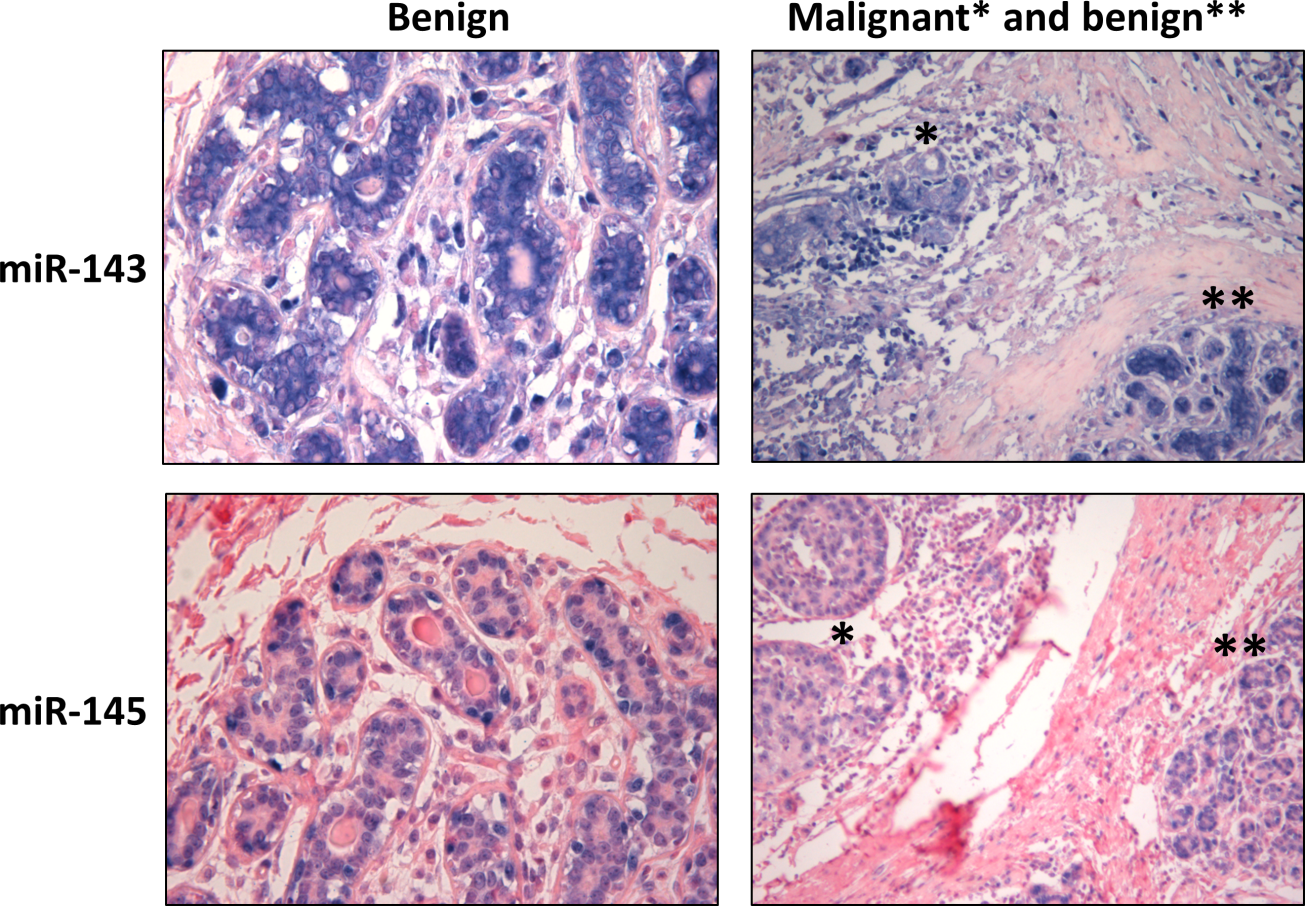
MiR-143 and miR-145 *in situ* hybridization staining pattern in breast tissue. Illustrative examples of miR-143 and miR-145 *in situ* hybridization staining pattern in benign breast tissue (x400 magnification) and adjacent benign and malignant breast tissue (x200 magnification).

The cellular and subcellular expression of miR-143 and miR-145 in benign and malignant breast tissue was evaluated in full histological slides of 16 tumors with adjacent benign breast tissue. MiR-143 ISH staining was mainly cytoplasmatic and found predominantly in luminal cells in benign breast tissue. Noteworthy, the staining intensity in benign breast ducts and lobuli was strong and homogenous, and appeared stronger in benign tissue compared to adjacent tumor tissue, also in the tumors with moderate to strong staining intensity (Fig 7). As in benign tissue, miR-143 was expressed in the cytoplasm of tumor cells and stromal fibroblasts. However, in the tumors with high staining intensity in stromal cells, the staining was both cytoplasmatic and nuclear.

In contrast to the cytoplasmatic staining pattern for miR-143, miR-145 was expressed in the nuclei and with the strongest staining intensity in the myoepithelial cells in benign breast tissue (Fig 7). MiR-145 staining intensity was strong in benign breast tissue and tended to be stronger in benign compared to malignant breast tissue, but the difference was not as clear as for miR-143 (Fig 7). Also in tumor cells and stromal fibroblasts, miR-145 staining was predominantly nuclear.

**Fig 8:**
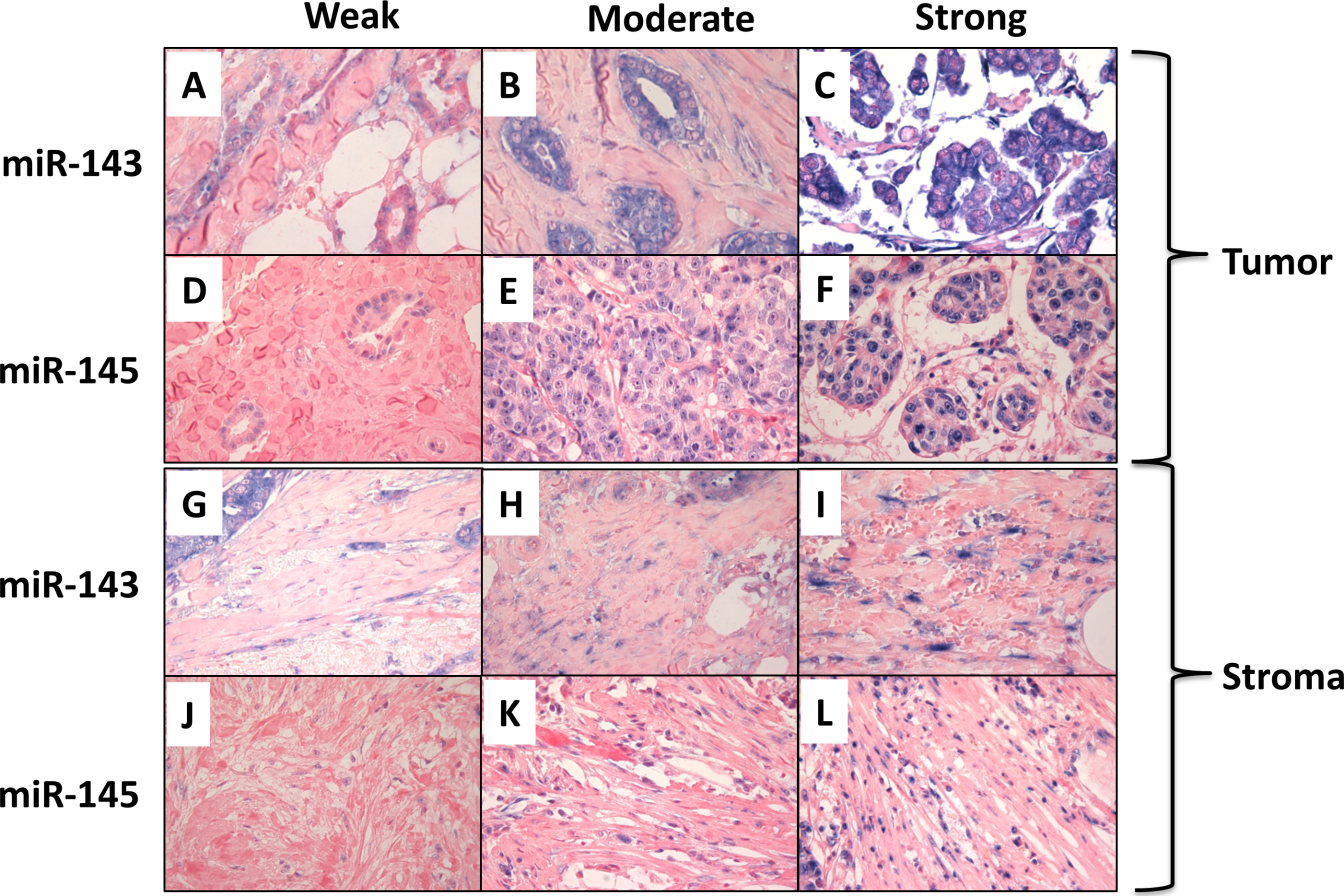
MiR-143 and miR-145 *in situ* hybridization staining intensities in tumor and stromal cells. *In situ* hybridization staining of miR-143 and miR-145 in tumor cells (panel A-C and panel D-F, respectively) and stromal fibroblasts (panel G-I and panel J-L, respectively) with examples of staining intensities corresponding to score weak, moderate and strong. Light microscope images, x400 magnification.

Tumor cells and stromal fibroblasts were scored for staining intensity as illustrated in Fig 8. Noteworthy, all 16 tumors had positive staining for both miR-143 and miR-145 in both tumor cells and fibroblasts. The mean staining intensity for miR-143 in tumor cells was 2.17 and in stromal fibroblasts 2.06 whereas mean miR-145 staining intensity was 2.10 in tumor cells and 1.69 in stromal fibroblasts. Using Spearman’s rho, the staining intensity in tumor cells and stromal fibroblasts was found to be positively correlated for both miR-143 (p = 0.006) and miR-145 (p = 0.006). Stromal ISH expression of miR-143 and miR-145 was also correlated (p = 0.049) (Table 6).

**Table 6:** Correlations of miR-143 and miR-145 *in situ* hybridization staining intensities in tumor and stromal cells.

	N = 16		miR-143		miR-145	
			Tumor	Stroma	Tumor	Stroma
**miR-143**	**Tumor**	**r**		0.657**	0.335	0.411
	**Stroma**	**r**	0.657**		0.275	0.499*
**miR-145**	**Tumor**	**r**	0.335	0.275		0.651**
	**Stroma**	**r**	0.411	0.499*	0.651**	

Spearman’s correlation, Spearman’s rho (r) presented in the table.

*P<0.05

**P<0.01.

## Discussion

This study characterizes the functional properties and expression pattern of the miRNA cluster miR-143 and miR-145 in BC.

Using microarray and PCR, we found a significant downregulation of miR-143 and miR-145 in malignant tumors compared to normal tissue, which is in line with previously published studies in breast cancer [29–31] and other tissues, especially colorectal carcinomas [32]. However, in our study the results were also verified by ISH, which added interesting data on the cellular and subcellular location of these miRNAs in benign and malignant breast tissue. Using ISH, we found the highest expression of miR-145 in myoepithelial cells in benign breast tissue. This is in line with other smaller studies using FISH or laser microdissection and PCR-analyses of miRNA expression [33, 34]. Further, we observed a distinct nuclear enrichment of mature miR-145, as has been previously reported in both breast and other tissues [34–36]. This is interesting, as the general dogma of miRNA biosynthesis and function involves post-transcriptional regulation of mRNA in the cytoplasm [37]. Although the nuclear functions of mature miRNAs remain elusive, there is growing evidence of specific miRNAs localized to the nucleus [38–40]. Park et al. suggest that nuclear miRNAs could uncover an entirely new role for this family of non-coding RNAs [40] where transportation across the nuclear membrane could regulate miRNA storage and function. Further, it is suggested that nuclear miRNA could be involved in post-transcriptional gene silencing via the nuclear RNA induced silencing complex (nRISC), transcriptional gene activation via recruitment of transcriptional activators, and influence splicing decisions at specific exons (alternative splicing) [41].

Using microarray and PCR, we found miR-145 to be significantly higher in ER+ tumors, which is in line with a previous study on lymph-node negative tumors where ER status was based on gene expression using microarray [42]. In our study, the expression of both miR-143 and miR-145 was elevated in the least aggressive tumor types, which is in line with the tumor suppressor functions described for these miRNAs in previous publications [15–19, 43]. Further, the expression of the two miRNAs seems to correlate in benign and malignant breast tissue and between tumor types, an observation that could partly be contributed to their transcriptional regulation, where the two miRNAs are located within the same chromosomal region and are transcribed as a cluster.

However, miRNA functions are complex. In our *in vitro* experiments, miR-143 was revealed to increase the proliferative capacity of the ER+ cell line MCF7 and the TN cell line MDA-MB-231, whilst having no significant effect on proliferation in the HER2+ cell line SK-BR-3. The effect was pronounced (Fig 2A and 2C), and reproduced in three biological replicates. This finding is in contrast to the majority of studies regarding the effect of miR-143 on proliferation [15–19]. Interestingly, we found that miR-143 had a significant inhibitory effect on cell invasion in all cell lines. The overexpression of miR-145 led to inhibition of both proliferation and invasion, which is in accordance with the general consensus [19, 20, 22–24, 44–51]. Noteworthy, the coexpression of miR-143 and miR-145 led to inhibition of proliferation and invasion, which is also described in previous publications [52–55]. At present, we do not have enough information to offer any explanatory model for the effects demonstrated by miR-143 in our proliferation studies. However, Dimitrova et al. describe stromal expression of the miR-143/145 cluster as a stimulator of neoangiogenesis, and a supporter of tumor expansion in lung [56]. Recently, Donnarumma et al. reported that the levels of miR-143 are increased in exosomes from cancer associated fibroblasts, and could promote breast cancer progression through exosome-mediated delivery of miRNA to breast cancer cells [57]. Estradiol has been published to downregulate expression of miR-143 in an ER-dependent manner in the ER+ cell line MCF7, whilst having no effect on miRNA levels in the TN cell line MDA-MB-231 [43]. Downregulation of miR-143 appears to contribute to the estradiol-mediated upregulation of bcl-2 and survivin. These observations underline that effects of signaling molecules, where miRNAs could be included, are highly dependent on the cellular context. Further, the stimulation of proliferation and, at the same time, inhibition of invasion, argue for several regulated target genes which could have opposing effects in tumorigenesis, as indeed demonstrated by the target genes for miR-143 listed in S1 Table.

Spizzo et al. verified miR-145 to directly target ER, and at the same time activate TP53, and in turn apoptosis [51]. These results may in part explain some of the inhibitory effects we observed in cell lines transfected with miR-145, but our experiments also demonstrated inhibition of proliferation and invasion in the TN *p53* mutant MDA-MB-231 and the ER- *p53* mutant SK-BR-3. These results argue for a different tumor suppressive pathway than described by Spizzo et al. However, the list of verified miR-145 target genes involved in tumor progression is wide-ranging (S2 Table), and the dominant active pathway(s) at any given time would be challenging to predict. S1 and S2 Tables present the validated mRNA targets for miR-143 and miR-145, based on searches using the online database miRTarBase [58].

Synergistic effects of miR-143 and miR-145 on BC cell proliferation have been demonstrated in MCF7 and MBA-MD-231 cells and in a mouse model using MCF7 xenografts [53]. Interestingly, both miRNAs, although not sharing sequence homology, were demonstrated to bind to the 3’-UTR region of HER3, the most potent effects were demonstrated by miR-145. Further, high HER3 expression could rescue the cells from the inhibitory effects of miR-143 and miR-145, underlining the importance of the cellular context and active signaling pathways on the net effect of miRNA up- or downregulation. Different results in different cell lines may be explained by miRNAs effects on target genes that are operating or driving the oncogenic phenotype in specific cell types where ER would be the classical example in ER+ BC. Noteworthy, miRNA-profiling of BC cells has demonstrated that miRNA-based clusters follow ER-based dichotomization of cell lines, indicating that a significant number of miRNAs may directly, or indirectly, be regulated by ER-signaling [59].

The results in this study, including the differential effects *in vitro* of miR-143 in BC cell lines, the effects of cotransfecting miR-143 and miR-145, and the nuclear enrichment of miR-145 in breast tissue, underline the complexity of miRNA regulation and function. Studies of miRNA expression in tissues and cancer types should be supplied by functional research of individual miRNAs where non-canonical effects of miRNAs are being explored in greater detail.

## Supporting information

S1 Table List of miR-143 target genes verified by reporter assay and western blot. (PDF)

S2 Table List of miR-145 target genes verified by reporter assay and western blot. (PDF)

## References

[1] DeSantis CE, Lin CC, Mariotto AB, Siegel RL, Stein KD, Kramer JL, et al Cancer treatment and survivorship statistics, 2014. CA Cancer J Clin. 2014;64(4):252–71. doi: 10.3322/caac.21235 . 24890451 10.3322/caac.21235

[2] Vuong D, Simpson PT, Green B, Cummings MC, Lakhani SR. Molecular classification of breast cancer. Virchows Arch. 2014;465(1):1–14. doi: 10.1007/s00428-014-1593-7 . 24878755 10.1007/s00428-014-1593-7

[3] Goldhirsch A, Wood WC, Coates AS, Gelber RD, Thurlimann B, Senn HJ, et al Strategies for subtypes—dealing with the diversity of breast cancer: highlights of the St. Gallen International Expert Consensus on the Primary Therapy of Early Breast Cancer 2011. Ann Oncol. 2011;22(8):1736–47. doi: 10.1093/annonc/mdr304 ; PubMed Central PMCID: PMCPMC3144634. 21709140 10.1093/annonc/mdr304 PMC3144634

[4] Perou CM, Sorlie T, Eisen MB, van de Rijn M, Jeffrey SS, Rees CA, et al Molecular portraits of human breast tumours. Nature. 2000;406(6797):747–52. doi: 10.1038/35021093 . 10963602 10.1038/35021093

[5] Masood S. Breast cancer subtypes: morphologic and biologic characterization. Womens Health (Lond). 2016;12(1):103–19. doi: 10.2217/whe.15.99 . 26756229 10.2217/whe.15.99 PMC5779568

[6] Yadav BS, Sharma SC, Chanana P, Jhamb S. Systemic treatment strategies for triple-negative breast cancer. World J Clin Oncol. 2014;5(2):125–33. doi: 10.5306/wjco.v5.i2.125 ; PubMed Central PMCID: PMCPMC4014784. 24829859 10.5306/wjco.v5.i2.125 PMC4014784

[7] Sotiriou C, Neo SY, McShane LM, Korn EL, Long PM, Jazaeri A, et al Breast cancer classification and prognosis based on gene expression profiles from a population-based study. Proc Natl Acad Sci U S A. 2003;100(18):10393–8. doi: 10.1073/pnas.1732912100 ; PubMed Central PMCID: PMCPMC193572. 12917485 10.1073/pnas.1732912100 PMC193572

[8] Jonas S, Izaurralde E. Towards a molecular understanding of microRNA-mediated gene silencing. Nat Rev Genet. 2015;16(7):421–33. doi: 10.1038/nrg3965 . 26077373 10.1038/nrg3965

[9] Winter J, Jung S, Keller S, Gregory RI, Diederichs S. Many roads to maturity: microRNA biogenesis pathways and their regulation. Nat Cell Biol. 2009;11(3):228–34. doi: 10.1038/ncb0309-228 . 19255566 10.1038/ncb0309-228

[10] Calin GA, Croce CM. MicroRNA signatures in human cancers. Nat Rev Cancer. 2006;6(11):857–66. doi: 10.1038/nrc1997 . 17060945 10.1038/nrc1997

[11] Naeini MM, Ardekani AM. Noncoding RNAs and Cancer. Avicenna J Med Biotechnol. 2009;1(2):55–70. ; PubMed Central PMCID: PMCPMC3558126. 23407615 PMC3558126

[12] Zhang C. MicroRNAs: role in cardiovascular biology and disease. Clin Sci (Lond). 2008;114(12):699–706. doi: 10.1042/CS20070211 . 18474027 10.1042/CS20070211

[13] Lu J, Getz G, Miska EA, Alvarez-Saavedra E, Lamb J, Peck D, et al MicroRNA expression profiles classify human cancers. Nature. 2005;435(7043):834–8. doi: 10.1038/nature03702 . 15944708 10.1038/nature03702

[14] Ruan K, Fang X, Ouyang G. MicroRNAs: novel regulators in the hallmarks of human cancer. Cancer Lett. 2009;285(2):116–26. doi: 10.1016/j.canlet.2009.04.031 . 19464788 10.1016/j.canlet.2009.04.031

[15] Liu J, Mao Y, Zhang D, Hao S, Zhang Z, Li Z, et al MiR-143 inhibits tumor cell proliferation and invasion by targeting STAT3 in esophageal squamous cell carcinoma. Cancer letters. 2016;373(1):97–108. doi: 10.1016/j.canlet.2016.01.023 . 26806810 10.1016/j.canlet.2016.01.023

[16] Xu YF, Li YQ, Guo R, He QM, Ren XY, Tang XR, et al Identification of miR-143 as a tumour suppressor in nasopharyngeal carcinoma based on microRNA expression profiling. Int J Biochem Cell Biol. 2015;61:120–8. doi: 10.1016/j.biocel.2015.02.006 . 25701793 10.1016/j.biocel.2015.02.006

[17] Wang Q, Cai J, Wang J, Xiong C, Zhao J. MiR-143 inhibits EGFR-signaling-dependent osteosarcoma invasion. Tumour Biol. 2014;35(12):12743–8. doi: 10.1007/s13277-014-2600-y . 25227664 10.1007/s13277-014-2600-y

[18] Xia H, Sun S, Wang B, Wang T, Liang C, Li G, et al miR-143 inhibits NSCLC cell growth and metastasis by targeting Limk1. Int J Mol Sci. 2014;15(7):11973–83. doi: 10.3390/ijms150711973 ; PubMed Central PMCID: PMCPMC4139824. 25003638 10.3390/ijms150711973 PMC4139824

[19] Zhang W, Wang Q, Yu M, Wu N, Wang H. MicroRNA-145 function as a cell growth repressor by directly targeting c-Myc in human ovarian cancer. Technology in cancer research & treatment. 2014;13(2):161–8. Epub 2013/08/08. doi: 10.7785/tcrt.2012.500367 . 23919393 10.7785/tcrt.2012.500367

[20] Wu J, Yin L, Jiang N, Guo WJ, Gu JJ, Chen M, et al MiR-145, a microRNA targeting ADAM17, inhibits the invasion and migration of nasopharyngeal carcinoma cells. Exp Cell Res. 2015;338(2):232–8. doi: 10.1016/j.yexcr.2015.08.006 . 26297956 10.1016/j.yexcr.2015.08.006

[21] Chen JJ, Cai WY, Liu XW, Luo QC, Chen G, Huang WF, et al Reverse Correlation between MicroRNA-145 and FSCN1 Affecting Gastric Cancer Migration and Invasion. PLoS One. 2015;10(5):e0126890 doi: 10.1371/journal.pone.0126890 ; PubMed Central PMCID: PMCPMC4444015. 26010149 10.1371/journal.pone.0126890 PMC4444015

[22] Qin J, Wang F, Jiang H, Xu J, Jiang Y, Wang Z. MicroRNA-145 suppresses cell migration and invasion by targeting paxillin in human colorectal cancer cells. Int J Clin Exp Pathol. 2015;8(2):1328–40. ; PubMed Central PMCID: PMCPMC4396207. 25973017 PMC4396207

[23] Larne O, Hagman Z, Lilja H, Bjartell A, Edsjo A, Ceder Y. miR-145 suppress the androgen receptor in prostate cancer cells and correlates to prostate cancer prognosis. Carcinogenesis. 2015;36(8):858–66. doi: 10.1093/carcin/bgv063 . 25969144 10.1093/carcin/bgv063

[24] Zhang Y, Yang X, Wu H, Zhou W, Liu Z. MicroRNA-145 inhibits migration and invasion via inhibition of fascin 1 protein expression in non-small-cell lung cancer cells. Mol Med Rep. 2015;12(4):6193–8. doi: 10.3892/mmr.2015.4163 . 26238532 10.3892/mmr.2015.4163

[25] Dumeaux V, Borresen-Dale AL, Frantzen JO, Kumle M, Kristensen VN, Lund E. Gene expression analyses in breast cancer epidemiology: the Norwegian Women and Cancer postgenome cohort study. Breast cancer research. 2008;10(1):R13 Epub 2008/02/15. doi: 10.1186/bcr1859 ; PubMed Central PMCID: PMCPmc2374969. 18271962 10.1186/bcr1859 PMC2374969

[26] Elston CW, Ellis IO. Pathological prognostic factors in breast cancer. I. The value of histological grade in breast cancer: experience from a large study with long-term follow-up. Histopathology. 1991;19(5):403–10. Epub 1991/11/01. . 1757079 10.1111/j.1365-2559.1991.tb00229.x

[27] Coates AS, Winer EP, Goldhirsch A, Gelber RD, Gnant M, Piccart-Gebhart M, et al Tailoring therapies—improving the management of early breast cancer: St Gallen International Expert Consensus on the Primary Therapy of Early Breast Cancer 2015. Ann Oncol. 2015;26(8):1533–46. Epub 2015/05/06. doi: 10.1093/annonc/mdv221 ; PubMed Central PMCID: PMCPmc4511219. 25939896 10.1093/annonc/mdv221 PMC4511219

[28] Vasconcelos I, Hussainzada A, Berger S, Fietze E, Linke J, Siedentopf F, et al The St. Gallen surrogate classification for breast cancer subtypes successfully predicts tumor presenting features, nodal involvement, recurrence patterns and disease free survival. Breast. 2016;29:181–5. Epub 2016/08/22. doi: 10.1016/j.breast.2016.07.016 . 27544822 10.1016/j.breast.2016.07.016

[29] Zheng T, Zhang X, Wang Y, Yu X. Predicting associations between microRNAs and target genes in breast cancer by bioinformatics analyses. Oncology letters. 2016;12(2):1067–73. Epub 2016/07/23. doi: 10.3892/ol.2016.4731 ; PubMed Central PMCID: PMCPmc4950656. 27446395 10.3892/ol.2016.4731 PMC4950656

[30] Iorio MV, Ferracin M, Liu C-G, Veronese A, Spizzo R, Sabbioni S, et al MicroRNA Gene Expression Deregulation in Human Breast Cancer. Cancer Research. 2005;65(16):7065–70. doi: 10.1158/0008-5472.CAN-05-1783 16103053 10.1158/0008-5472.CAN-05-1783

[31] Navon R, Wang H, Steinfeld I, Tsalenko A, Ben-Dor A, Yakhini Z. Novel Rank-Based Statistical Methods Reveal MicroRNAs with Differential Expression in Multiple Cancer Types. PLOS ONE. 2009;4(11):e8003 doi: 10.1371/journal.pone.0008003 19946373 10.1371/journal.pone.0008003 PMC2777376

[32] Michael MZ, O' Connor SM, van Holst Pellekaan NG, Young GP, James RJ. Reduced Accumulation of Specific MicroRNAs in Colorectal Neoplasia. Molecular Cancer Research. 2003;1(12):882–91. 14573789

[33] Bockmeyer CL, Christgen M, Muller M, Fischer S, Ahrens P, Langer F, et al MicroRNA profiles of healthy basal and luminal mammary epithelial cells are distinct and reflected in different breast cancer subtypes. Breast cancer research and treatment. 2011;130(3):735–45. Epub 2011/03/17. doi: 10.1007/s10549-010-1303-3 . 21409395 10.1007/s10549-010-1303-3

[34] Sempere LF, Christensen M, Silahtaroglu A, Bak M, Heath CV, Schwartz G, et al Altered MicroRNA expression confined to specific epithelial cell subpopulations in breast cancer. Cancer Res. 2007;67(24):11612–20. Epub 2007/12/20. doi: 10.1158/0008-5472.CAN-07-5019 . 18089790 10.1158/0008-5472.CAN-07-5019

[35] Sangiao-Alvarellos S, Manfredi-Lozano M, Ruiz-Pino F, León S, Morales C, Cordido F, et al Testicular expression of the Lin28/let-7 system: Hormonal regulation and changes during postnatal maturation and after manipulations of puberty. Scientific Reports. 2015;5:15683 doi: 10.1038/srep15683. http://www.nature.com/articles/srep15683#supplementary-information. 26494358 10.1038/srep15683 PMC4616161

[36] Liu Y, Wu C, Wang Y, Wen S, Wang J, Chen Z, et al Expression of miR-224, miR-145, and their putative target ADAM17 in hepatocellular carcinoma. Acta Biochimica et Biophysica Sinica. 2014;46(8):720–2. doi: 10.1093/abbs/gmu052 24969373 10.1093/abbs/gmu052

[37] Ha M, Kim VN. Regulation of microRNA biogenesis. Nat Rev Mol Cell Biol. 2014;15(8):509–24. doi: 10.1038/nrm3838 . 25027649 10.1038/nrm3838

[38] Li ZF, Liang YM, Lau PN, Shen W, Wang DK, Cheung WT, et al Dynamic localisation of mature microRNAs in Human nucleoli is influenced by exogenous genetic materials. PLoS One. 2013;8(8):e70869 doi: 10.1371/journal.pone.0070869 ; PubMed Central PMCID: PMCPMC3735495. 23940654 10.1371/journal.pone.0070869 PMC3735495

[39] Wei Y, Li L, Wang D, Zhang CY, Zen K. Importin 8 regulates the transport of mature microRNAs into the cell nucleus. J Biol Chem. 2014;289(15):10270–5. doi: 10.1074/jbc.C113.541417 ; PubMed Central PMCID: PMCPMC4036152. 24596094 10.1074/jbc.C113.541417 PMC4036152

[40] Park CW, Zeng Y, Zhang X, Subramanian S, Steer CJ. Mature microRNAs identified in highly purified nuclei from HCT116 colon cancer cells. RNA Biol. 2010;7(5):606–14. doi: 10.4161/rna.7.5.13215 ; PubMed Central PMCID: PMCPMC3073257. 20864815 10.4161/rna.7.5.13215 PMC3073257

[41] Roberts TC. The MicroRNA Biology of the Mammalian Nucleus. Molecular therapy Nucleic acids. 2014;3:e188 Epub 2014/08/20. doi: 10.1038/mtna.2014.40 ; PubMed Central PMCID: PMCPmc4221600. 25137140 10.1038/mtna.2014.40 PMC4221600

[42] Foekens JA, Sieuwerts AM, Smid M, Look MP, de Weerd V, Boersma AW, et al Four miRNAs associated with aggressiveness of lymph node-negative, estrogen receptor-positive human breast cancer. Proc Natl Acad Sci U S A. 2008;105(35):13021–6. Epub 2008/08/30. doi: 10.1073/pnas.0803304105 ; PubMed Central PMCID: PMCPmc2529088. 18755890 10.1073/pnas.0803304105 PMC2529088

[43] Yu X, Zhang X, Dhakal IB, Beggs M, Kadlubar S, Luo D. Induction of cell proliferation and survival genes by estradiol-repressed microRNAs in breast cancer cells. BMC cancer. 2012;12:29 Epub 2012/01/21. doi: 10.1186/1471-2407-12-29 ; PubMed Central PMCID: PMCPmc3274428. 22260523 10.1186/1471-2407-12-29 PMC3274428

[44] Liu Y, Wu C, Wang Y, Wen S, Wang J, Chen Z, et al MicroRNA-145 inhibits cell proliferation by directly targeting ADAM17 in hepatocellular carcinoma. Oncol Rep. 2014;32(5):1923–30. doi: 10.3892/or.2014.3424 . 25174729 10.3892/or.2014.3424

[45] Sachdeva M, Mo Y-Y. MicroRNA-145 Suppresses Cell Invasion and Metastasis by Directly Targeting Mucin 1. Cancer Research. 2010;70(1):378–87. doi: 10.1158/0008-5472.CAN-09-2021 19996288 10.1158/0008-5472.CAN-09-2021 PMC2805032

[46] Sachdeva M, Mo YY. MicroRNA-145 suppresses cell invasion and metastasis by directly targeting mucin 1. Cancer Res. 2010;70(1):378–87. doi: 10.1158/0008-5472.CAN-09-2021 ; PubMed Central PMCID: PMCPMC2805032. 19996288 10.1158/0008-5472.CAN-09-2021 PMC2805032

[47] Fan L, Wu Q, Xing X, Wei Y, Shao Z. MicroRNA-145 targets vascular endothelial growth factor and inhibits invasion and metastasis of osteosarcoma cells. Acta Biochim Biophys Sin (Shanghai). 2012;44(5):407–14. doi: 10.1093/abbs/gms019 . 22472569 10.1093/abbs/gms019

[48] Cui SY, Wang R, Chen LB. MicroRNA-145: a potent tumour suppressor that regulates multiple cellular pathways. Journal of cellular and molecular medicine. 2014;18(10):1913–26. Epub 2014/08/16. doi: 10.1111/jcmm.12358 ; PubMed Central PMCID: PMCPmc4244007. 25124875 10.1111/jcmm.12358 PMC4244007

[49] Wang S, Bian C, Yang Z, Bo Y, Li J, Zeng L, et al miR-145 inhibits breast cancer cell growth through RTKN. International journal of oncology. 2009;34(5):1461–6. Epub 2009/04/11. . 19360360

[50] Cho WC, Chow AS, Au JS. MiR-145 inhibits cell proliferation of human lung adenocarcinoma by targeting EGFR and NUDT1. RNA Biol. 2011;8(1):125–31. . 21289483 10.4161/rna.8.1.14259

[51] Spizzo R, Nicoloso MS, Lupini L, Lu Y, Fogarty J, Rossi S, et al miR-145 participates with TP53 in a death-promoting regulatory loop and targets estrogen receptor-alpha in human breast cancer cells. Cell death and differentiation. 2010;17(2):246–54. Epub 2009/09/05. doi: 10.1038/cdd.2009.117 ; PubMed Central PMCID: PMCPmc3648637. 19730444 10.1038/cdd.2009.117 PMC3648637

[52] Su J, Liang H, Yao W, Wang N, Zhang S, Yan X, et al MiR-143 and MiR-145 regulate IGF1R to suppress cell proliferation in colorectal cancer. PLoS One. 2014;9(12):e114420 doi: 10.1371/journal.pone.0114420 ; PubMed Central PMCID: PMCPMC4256231. 25474488 10.1371/journal.pone.0114420 PMC4256231

[53] Yan X, Chen X, Liang H, Deng T, Chen W, Zhang S, et al miR-143 and miR-145 synergistically regulate ERBB3 to suppress cell proliferation and invasion in breast cancer. Molecular cancer. 2014;13:220 Epub 2014/09/25. doi: 10.1186/1476-4598-13-220 ; PubMed Central PMCID: PMCPmc4181414. 25248370 10.1186/1476-4598-13-220 PMC4181414

[54] Yoshino H, Enokida H, Itesako T, Kojima S, Kinoshita T, Tatarano S, et al Tumor-suppressive microRNA-143/145 cluster targets hexokinase-2 in renal cell carcinoma. Cancer Sci. 2013;104(12):1567–74. doi: 10.1111/cas.12280 . 24033605 10.1111/cas.12280 PMC7653528

[55] Zhang X, Dong Y, Ti H, Zhao J, Wang Y, Li T, et al Down-regulation of miR-145 and miR-143 might be associated with DNA methyltransferase 3B overexpression and worse prognosis in endometrioid carcinomas. Hum Pathol. 2013;44(11):2571–80. doi: 10.1016/j.humpath.2013.07.002 . 24071015 10.1016/j.humpath.2013.07.002

[56] Dimitrova N, Gocheva V, Bhutkar A, Resnick R, Jong RM, Miller KM, et al Stromal Expression of miR-143/145 Promotes Neoangiogenesis in Lung Cancer Development. Cancer Discov. 2016;6(2):188–201. doi: 10.1158/2159-8290.CD-15-0854 ; PubMed Central PMCID: PMCPMC4744583. 26586766 10.1158/2159-8290.CD-15-0854 PMC4744583

[57] Donnarumma E, Fiore D, Nappa M, Roscigno G, Adamo A, Iaboni M, et al Cancer-associated fibroblasts release exosomal microRNAs that dictate an aggressive phenotype in breast cancer. Oncotarget. 2017;8(12):19592–608. doi: 10.18632/oncotarget.14752 ; PubMed Central PMCID: PMCPMC5386708. 28121625 10.18632/oncotarget.14752 PMC5386708

[58] Chou CH, Chang NW, Shrestha S, Hsu SD, Lin YL, Lee WH, et al miRTarBase 2016: updates to the experimentally validated miRNA-target interactions database. Nucleic Acids Res. 2016;44(D1):D239–47. doi: 10.1093/nar/gkv1258 ; PubMed Central PMCID: PMCPMC4702890. 26590260 10.1093/nar/gkv1258 PMC4702890

[59] Riaz M, van Jaarsveld MT, Hollestelle A, Prager-van der Smissen WJ, Heine AA, Boersma AW, et al miRNA expression profiling of 51 human breast cancer cell lines reveals subtype and driver mutation-specific miRNAs. Breast cancer research. 2013;15(2):R33 Epub 2013/04/23. doi: 10.1186/bcr3415 ; PubMed Central PMCID: PMCPmc3672661. 23601657 10.1186/bcr3415 PMC3672661

