# Supplementary material for: Expression and function of the miR-143/145 cluster in vitro and in vivo in human breast cancer
Source: PLoS One. 2017 Oct 26;12(10):e0186658. doi: 10.1371/journal.pone.0186658 (PMC5657998; doi:10.1371/journal.pone.0186658)
Supplement: S1 Table — (PDF) [file pone.0186658.s001.pdf]

S1 table. List of miR-143 target genes verified by reporter assay and western blot.

Target gene	***Function***
KRAS	A well-studied proto-oncogene associated with various cancers
MYO6	Knockdown of MYO6 markedly reduces cell viability and colony formation, as well as suppresses cell cycle progression in breast cancer cells (1)
DNMT3A	Overexpression is associated with tumorigenesis in breast, liver, leukemia, colon, and prostate cancers (2)
SERPINE1	Regulates invasion and metastasis by upregulating MMP-13 expression in human osteosarcoma (3)
FNDC3B	FNDC3B is an important oncogenic driver gene of the 3q amplicon (4)
MAPK7	Promotes breast cancer cell invasion and metastasis (5)
FSCN1	Plays an oncogenic role in non small cell lung cancer by activating the transcriptional activity of the YAP/TEAD complex (6)
HK2	Overexpressed in tumors and contributes to aerobic glycolysis, and thus plays an important role in the Warburg effect (7)
PTGS2	Involved in promoting metastasis and in delay of tumor progression in poorly differentiated metastatic breast cancer cells (8)
JAG1	Overexpression has been correlated with poor overall breast cancer survival and an enhancement of tumor proliferation in adrenocortical carcinoma (9)
AKT1	Associated with increased local tumor growth in breast cancer (10)
BCL2	A potent anti-apoptotic protein involved in various cancers, including breast cancer (11)
MMP13	Reported to be expressed at high level in various malignant tumors and is associated with metastasis of tumors, including breast cancer (12)
SDC1	Inhibits osteoclast functions in metastatic breast cancer to the bone through up-regulation of osteoprotegerin (13)
RREB1	RREB-1 may play a role in Ras and Raf signal transduction in medullary thyroid cancer and other cells (14)
CD44	CD44 induces FOXP3 expression and is related with favorable outcome in breast carcinoma (15)
KLF5	Proto-oncogene [2]
BRAF	B-Raf is a member of the Raf kinase family and plays a role in regulating the MAP kinase/ERKs signaling pathway, which affects cell division and differentiation (16)
IGF1R	Implicated in several cancers, and is linked to acquired therapeutic resistance (17, 18)
NR2C2	Suppresses ER function via protein-protein interaction in breast cancer cells (19)
MACC1	Elevated in breast cancer, and high levels are linked to poor survival (20)
DDX6	Responsible for radio- and chemoresistance in glioblastoma (21)
LIMK1	LIMK1 activity in both the cytoplasmic and nuclear compartments of breast tissue promotes breast cancer progression (22)

References

1. Wang H, Wang B, Zhu W, Yang Z. Lentivirus-Mediated Knockdown of Myosin VI Inhibits Cell Proliferation of Breast Cancer Cell. Cancer Biother Radiopharm. 2015;30(8):330-5.
2. Deivendran S, Marzook H, Santhoshkumar TR, Kumar R, Pillai MR. Metastasis-associated protein 1 is an upstream regulator of DNMT3a and stimulator of insulin-growth factor binding protein-3 in breast cancer. Sci Rep. 2017;7:44225.
3. Hirahata M, Osaki M, Kanda Y, Sugimoto Y, Yoshioka Y, Kosaka N, et al. PAI-1, a target gene of miR-143, regulates invasion and metastasis by upregulating MMP-13 expression of human osteosarcoma. Cancer Med. 2016;5(5):892-902.
4. Cai C, Rajaram M, Zhou X, Liu Q, Marchica J, Li J, et al. Activation of multiple cancer pathways and tumor maintenance function of the 3q amplified oncogene FNDC3B. Cell Cycle. 2012;11(9):1773-81.
5. Liu F, Zhang H, Song H. Upregulation of MEK5 by Stat3 promotes breast cancer cell invasion and metastasis. Oncol Rep. 2017;37(1):83-90.
6. Liang Z, Wang Y, Shen Z, Teng X, Li X, Li C, et al. Fascin 1 promoted the growth and migration of non-small cell lung cancer cells by activating YAP/TEAD signaling. Tumour Biol. 2016;37(8):10909-15.
7. Jiang S, Zhang LF, Zhang HW, Hu S, Lu MH, Liang S, et al. A novel miR-155/miR-143 cascade controls glycolysis by regulating hexokinase 2 in breast cancer cells. EMBO J. 2012;31(8):1985-98.
8. Stasinopoulos I, O'Brien DR, Wildes F, Glunde K, Bhujwalla ZM. Silencing of cyclooxygenase-2 inhibits metastasis and delays tumor onset of poorly differentiated metastatic breast cancer cells. Mol Cancer Res. 2007;5(5):435-42.
9. Dickson BC, Mulligan AM, Zhang H, Lockwood G, O'Malley FP, Egan SE, et al. High-level JAG1 mRNA and protein predict poor outcome in breast cancer. Mod Pathol. 2007;20(6):685-93.
10. Riggio M, Perrone MC, Polo ML, Rodriguez MJ, May M, Abba M, et al. AKT1 and AKT2 isoforms play distinct roles during breast cancer progression through the regulation of specific downstream proteins. Sci Rep. 2017;7:44244.
11. Hwang KT, Han W, Kim J, Moon HG, Oh S, Song YS, et al. Prognostic Influence of BCL2 on Molecular Subtypes of Breast Cancer. J Breast Cancer. 2017;20(1):54-64.
12. Xue J, Chen Z, Gu X, Zhang Y, Zhang W. MicroRNA-148a inhibits migration of breast cancer cells by targeting MMP-13. Tumour Biol. 2016;37(2):1581-90.
13. Benad-Mehner P, Thiele S, Rachner TD, Gobel A, Rauner M, Hofbauer LC. Targeting syndecan-1 in breast cancer inhibits osteoclast functions through up-regulation of osteoprotegerin. J Bone Oncol. 2014;3(1):18-24.
14. Thiagalingam A, De Bustros A, Borges M, Jasti R, Compton D, Diamond L, et al. RREB-1, a novel zinc finger protein, is involved in the differentiation response to Ras in human medullary thyroid carcinomas. Mol Cell Biol. 1996;16(10):5335-45.
15. Sanmartin E, Ortiz-Martinez F, Pomares-Navarro E, Garcia-Martinez A, Rodrigo-Banos M, Garcia-Escolano M, et al. CD44 induces FOXP3 expression and is related with favorable outcome in breast carcinoma. Virchows Arch. 2017;470(1):81-90.
16. Daum G, Eisenmann-Tappe I, Fries HW, Troppmair J, Rapp UR. The ins and outs of Raf kinases. Trends Biochem Sci. 1994;19(11):474-80.
17. Warshamana-Greene GS, Litz J, Buchdunger E, Garcia-Echeverria C, Hofmann F, Krystal GW. The insulin-like growth factor-I receptor kinase inhibitor, NVP-ADW742, sensitizes small cell lung cancer cell lines to the effects of chemotherapy. Clin Cancer Res. 2005;11(4):1563-71.
18. Jones HE, Goddard L, Gee JM, Hiscox S, Rubini M, Barrow D, et al. Insulin-like growth factor-I receptor signalling and acquired resistance to gefitinib (ZD1839; Iressa) in human breast and prostate cancer cells. Endocr Relat Cancer. 2004;11(4):793-814.
19. Shyr CR, Hu YC, Kim E, Chang C. Modulation of estrogen receptor-mediated transactivation by orphan receptor TR4 in MCF-7 cells. The Journal of biological chemistry. 2002;277(17):14622-8.

20. Tan W, Xie X, Li L, Tang H, Ye X, Chen L, et al. Diagnostic and prognostic value of serum MACC1 in breast cancer patients. Oncotarget. 2016;7(51):84408-15.
21. Cho YJ, Kang W, Kim SH, Sa JK, Kim N, Paddison PJ, et al. Involvement of DDX6 gene in radio- and chemoresistance in glioblastoma. International journal of oncology. 2016;48(3):1053-62.
22. McConnell BV, Koto K, Gutierrez-Hartmann A. Nuclear and cytoplasmic LIMK1 enhances human breast cancer progression. Molecular cancer. 2011;10:75.
