# Supplementary material for: Expression and function of the miR-143/145 cluster in vitro and in vivo in human breast cancer
Source: PLoS One. 2017 Oct 26;12(10):e0186658. doi: 10.1371/journal.pone.0186658 (PMC5657998; doi:10.1371/journal.pone.0186658)
Supplement: S2 Table — (PDF) [file pone.0186658.s002.pdf]

S2 table. List of miR-145 target genes verified by reporter assay and western blot.

Target gene	*Function in disease*
BNIP3	Bnip3 is a pro-apoptotic BH3-only protein which is associated with mitochondrial dysfunction and cell death (1)
KLF5	Proto-oncogene (2)
SOX2	Stimulates cell proliferation, migration, invasion, and tumor metastasis in various types of human malignancies (3)
KLF4	Group of transcriptional regulators that have recently been identified to exhibit tumor-suppressive functionsin various gastrointestinal cancers (4)
MUC1	Affects cancer progression in lung adenocarcinoma, and aberrant expression has been correlated with poor tumor differentiation and impaired prognosis (5)
MYO6	Knockdown of MYO6 markedly reduces cell viability and colony formation, as well as suppresses cell cycle progression in breast cancer cells (6)
CDKN1A	Significantly correlated with lymph node metastasis, distant metastases, advanced TNM stage, depth of invasion, and overall survival in gastric cancer (7)
ITGB8	High expression levels are associated with highly angiogenic and poorly invasive glioblastoma tumors. Low expression correlates with highly invasive, but poorly angiogenic tumors (8)
STAT1	A transcription factor involved in upregulating genes due to signals by either type I, type II, or type III interferons (9)
YES1	A proto-oncogene and a central mediator of cell growth in malignant mesothelioma cells (10)
IRS1	Plays a key role in transmitting signals from the insulin and insulin-like growth factor-1 (IGF-1) receptors to the PI3K/Akt and MAP kinase/ERKs signaling pathway (11)
VEGFA	Stimulates angiogenesis
HOXA9	A transcription factor downregulated in cervical cancer cells and its' restoration decreases proliferation, migration and expression of EMT transition genes (12)
EGFR	Growth factor receptor. Overexpression is associated with numerous cancers
FSCN1	Plays an oncogenic role in non small cell lung cancer by activating the transcriptional activity of the YAP/TEAD complex (13)
MYC	Amplification of Myc occurs in several cancer types, including breast, colorectal, pancreatic, gastric, and uterine cancers (14)
FLI1	Overexpression is associated with adverse prognosis of endometrial cancer (15)
DFFA	Apoptotic regulator (16)
POU5F1	Exhibits a distinctive expression pattern of different spliced variants in different types of breast cancer (17)
IGF1R	Implicated in several cancers, and is linked to acquired therapeutic resistance (18, 19)
ROBO2	Downregulated in prostate cancer, and is a candidate tumor suppressor gene (20)
SRGAP1	Downregulates ROBO2 (21)
EIF4E	Knockdown of eIF4E suppresses cell proliferation, invasion and enhances cisplatin cytotoxicity in human ovarian cancer cells (22)
CDK4	Dysregulation of cyclin-dependent kinases are greatly associated with cancer (23)
SERPINE1	Regulates invasion and metastasis by upregulating MMP-13 expression of human osteosarcoma (24)
SWAP70	SWAP70, actin-binding protein, function as an oncogene targeting tumor-suppressive miR-145 in prostate cancer (25)
JAG1	Overexpression has been correlated with poor overall breast cancer survival and an enhancement of tumor proliferation in adrenocortical carcinoma (26)
NEDD9	NEDD9 is an intermediate in a number of important signaling pathways relevant to the cellular processes of proliferation, survival, migration, and others (27)

PAK4	Activated Pak4 expression correlates with poor prognosis in several cancers
DDX17	A member of the DEAD-box RNA helicase family, and overexpression enhances malignant migration and invasion of glioma cells by repressing expression of Beclin1 (28)
NRAS	RAS mutations are frequently found among acute myeloid leukemia patients (AML), and other cancers, generating a constitutively active signaling protein changing cellular proliferation, differentiation and apoptosis (29)
ILK	Anti-integrin-linked kinase (ILK) therapies result in aberrant mitosis including altered mitotic spindle organization, centrosome declustering and mitotic arrest (30). Overexpression of ILK1 in breast cancer associates with poor prognosis (31)
ADAM17	ADAM17 in tumor associated leukocytes regulates inflammatory mediators and promotes mammary tumor formation (32)
CDH2	Cadherins associate with distinct stem cell-related transcription factors to coordinate the maintenance of stemness in triple-negative breast cancer (33)
RTKN	Links the Rho signal to nuclear factor-κB (NF-κB) activation, leading to increased cell survival by transactivating antiapoptotic genes downstream of NF-κB (34)
EPAS1	Regulators of hypoxic adaptive responses at both cellular and organismal levels (35)
ETS1	Ets1 is primarily expressed in triple-negative breast cancers, and contributes to the acquisition of cancer cell invasiveness, EMT (epithelial-to-mesenchymal transition), development of drug resistance and angiogenesis (36)
RREB1	RREB-1 may play a role in Ras and Raf signal transduction in medullary thyroid cancer and other cells (37)
CD44	CD44 induces FOXP3 expression and is associated with favorable outcome in breast carcinoma (38)
BRAF	B-Raf is a member of the Raf kinase family and plays a role in regulating the MAP kinase/ERKs signaling pathway, which affects cell division and differentiation (39)
SOX9	Involved in regulating hormone-dependent breast cancer cell proliferation (40)
SMAD3	Mediates the signals from the transforming growth factor beta (TGF-β) superfamily ligands that regulate cell proliferation, differentiation and death (41)
TGFBR2	High levels of TβRII are associated with lymph node metastasis, higher tumor stage, and poorer 5-year disease-free survival in breast cancer (42)
CTNND1	p120 is indispensable for the ability of HER2/ErbB2 to promote invasiveness in breast cancer cell lines (43)
SP1	Transcription of ER alpha is dependent upon the expression of members of the transcription factor Sp1 family (44)
TNFSF13	A cytokine that promotes breast tumor growth and metastasis, and is associated with aggressive basal breast cancer (45)
CDK6	Cells overexpressing CDK6 accumulates high levels of p53 and p130 (46)
DDX6	Responsible for radio- and chemoresistance in glioblastoma (47)
ADD3	Plays an important role in biliary atresia pathogenesis (48)
HMGA2	Elevated HMGA2 expression is associated with cancer aggressiveness and predicts poor outcome in breast cancer (49)
ROCK1	Linked to gender-related modulation in colorectal cancer susceptibility (50)
E2F3	Silencing of E2F3 suppresses tumor growth of HER2+ breast cancer cells by restricting mitosis (51)
SP7	Osterix (SP7), a transcription factor for osteoblast differentiation, mediates antitumor activity in murine osteosarcoma (52)
HDAC11	HDAC11 controls molecular mechanism promoting neuroblastoma through epigenetic suppression of the BMP4 gene, and is a novel drug target in carcinomas (53)

References

1. Quinsay MN, Thomas RL, Lee Y, Gustafsson AB. Bnip3-mediated mitochondrial autophagy is independent of the mitochondrial permeability transition pore. Autophagy. 2010;6(7):855-62.
2. Jiang Z, Zhang Y, Cao R, Li L, Zhong K, Chen Q, et al. MiR-5195-3p inhibits proliferation and invasion of human bladder cancer cells by directly targeting oncogene KLF5. Oncol Res. 2017.
3. Chen Y, Huang Y, Zhu L, Chen M, Huang Y, Zhang J, et al. SOX2 inhibits metastasis in gastric cancer. J Cancer Res Clin Oncol. 2016;142(6):1221-30.
4. Xiu DH, Chen Y, Liu L, Yang HS, Liu GF. Tumor-suppressive role of Kruppel-like factor 4 (KLF-4) in colorectal cancer. Genet Mol Res. 2017;16(1).
5. Lappi-Blanco E, Makinen JM, Lehtonen S, Karvonen H, Sormunen R, Laitakari K, et al. Mucin-1 correlates with survival, smoking status, and growth patterns in lung adenocarcinoma. Tumour Biol. 2016;37(10):13811-20.
6. Wang H, Wang B, Zhu W, Yang Z. Lentivirus-Mediated Knockdown of Myosin VI Inhibits Cell Proliferation of Breast Cancer Cell. Cancer Biother Radiopharm. 2015;30(8):330-5.
7. Leisibach P, Schneiter D, Soltermann A, Yamada Y, Weder W, Jungraithmayr W. Prognostic value of immunohistochemical markers in malignant thymic epithelial tumors. J Thorac Dis. 2016;8(9):2580-91.
8. Tchaicha JH, Reyes SB, Shin J, Hossain MG, Lang FF, McCarty JH. Glioblastoma angiogenesis and tumor cell invasiveness are differentially regulated by beta8 integrin. Cancer Res. 2011;71(20):6371-81.
9. Katze MG, He Y, Gale M, Jr. Viruses and interferon: a fight for supremacy. Nat Rev Immunol. 2002;2(9):675-87.
10. Sato A, Sekine M, Virgona N, Ota M, Yano T. Yes is a central mediator of cell growth in malignant mesothelioma cells. Oncol Rep. 2012;28(5):1889-93.
11. Copps KD, White MF. Regulation of insulin sensitivity by serine/threonine phosphorylation of insulin receptor substrate proteins IRS1 and IRS2. Diabetologia. 2012;55(10):2565-82.
12. Alvarado-Ruiz L, Martinez-Silva MG, Torres-Reyes LA, Pina-Sanchez P, Ortiz-Lazareno P, Bravo-Cuellar A, et al. HOXA9 is Underexpressed in Cervical Cancer Cells and its Restoration Decreases Proliferation, Migration and Expression of Epithelial-to-Mesenchymal Transition Genes. Asian Pac J Cancer Prev. 2016;17(3):1037-47.
13. Liang Z, Wang Y, Shen Z, Teng X, Li X, Li C, et al. Fascin 1 promoted the growth and migration of non-small cell lung cancer cells by activating YAP/TEAD signaling. Tumour Biol. 2016;37(8):10909-15.
14. Chen Y, McGee J, Chen X, Doman TN, Gong X, Zhang Y, et al. Identification of druggable cancer driver genes amplified across TCGA datasets. PLoS One. 2014;9(5):e98293.
15. Song W, Zhang T, Li W, Mu R, Zhang L, Li Y, et al. Overexpression of Fli-1 is associated with adverse prognosis of endometrial cancer. Cancer Invest. 2015;33(9):469-75.
16. Abel F, Sjoberg RM, Ejeskar K, Krona C, Martinsson T. Analyses of apoptotic regulators CASP9 and DFFA at 1P36.2, reveal rare allele variants in human neuroblastoma tumours. Br J Cancer. 2002;86(4):596-604.
17. Soheili S, Asadi MH, Farsinejad A. Distinctive expression pattern of OCT4 variants in different types of breast cancer. Cancer Biomark. 2017;18(1):69-76.
18. Warshamana-Greene GS, Litz J, Buchdunger E, Garcia-Echeverria C, Hofmann F, Krystal GW. The insulin-like growth factor-I receptor kinase inhibitor, NVP-ADW742, sensitizes small cell lung cancer cell lines to the effects of chemotherapy. Clin Cancer Res. 2005;11(4):1563-71.
19. Jones HE, Goddard L, Gee JM, Hiscox S, Rubini M, Barrow D, et al. Insulin-like growth factor-I receptor signalling and acquired resistance to gefitinib (ZD1839; Iressa) in human breast and prostate cancer cells. Endocr Relat Cancer. 2004;11(4):793-814.
20. Choi YJ, Yoo NJ, Lee SH. Down-regulation of ROBO2 expression in prostate cancers. Pathol Oncol Res. 2014;20(3):517-9.
21. Koo S, Martin G, Toussaint LG. MicroRNA-145 Promotes the Phenotype of Human Glioblastoma Cells Selected for Invasion. Anticancer Res. 2015;35(6):3209-15.

22. Wan J, Shi F, Xu Z, Zhao M. Knockdown of eIF4E suppresses cell proliferation, invasion and enhances cisplatin cytotoxicity in human ovarian cancer cells. International journal of oncology. 2015;47(6):2217-25.
23. O'Leary B, Finn RS, Turner NC. Treating cancer with selective CDK4/6 inhibitors. Nat Rev Clin Oncol. 2016;13(7):417-30.
24. Hirahata M, Osaki M, Kanda Y, Sugimoto Y, Yoshioka Y, Kosaka N, et al. PAI-1, a target gene of miR-143, regulates invasion and metastasis by upregulating MMP-13 expression of human osteosarcoma. Cancer Med. 2016;5(5):892-902.
25. Chiyomaru T, Tatarano S, Kawakami K, Enokida H, Yoshino H, Nohata N, et al. SWAP70, actin-binding protein, function as an oncogene targeting tumor-suppressive miR-145 in prostate cancer. Prostate. 2011;71(14):1559-67.
26. Dickson BC, Mulligan AM, Zhang H, Lockwood G, O'Malley FP, Egan SE, et al. High-level JAG1 mRNA and protein predict poor outcome in breast cancer. Mod Pathol. 2007;20(6):685-93.
27. Singh M, Cowell L, Seo S, O'Neill G, Golemis E. Molecular basis for HEF1/NEDD9/Cas-L action as a multifunctional co-ordinator of invasion, apoptosis and cell cycle. Cell Biochem Biophys. 2007;48(1):54-72.
28. Zhang Z, Tian H, Miao Y, Feng X, Li Y, Wang H, et al. Upregulation of p72 Enhances Malignant Migration and Invasion of Glioma Cells by Repressing Beclin1 Expression. Biochemistry (Mosc). 2016;81(6):574-82.
29. Brendel C, Teichler S, Millahn A, Stiewe T, Krause M, Stabla K, et al. Oncogenic NRAS Primes Primary Acute Myeloid Leukemia Cells for Differentiation. PLoS One. 2015;10(4):e0123181.
30. Duminuco R, Noble JW, Goody J, Sharma M, Ksander BR, Roskelley CD, et al. Integrin-linked kinase regulates senescence in an Rb-dependent manner in cancer cell lines. Cell Cycle. 2015;14(18):2924-37.
31. Yang HJ, Zheng YB, Ji T, Ding XF, Zhu C, Yu XF, et al. Overexpression of ILK1 in breast cancer associates with poor prognosis. Tumour Biol. 2013;34(6):3933-8.
32. Bohrer LR, Chaffee TS, Chuntova P, Brady NJ, Witschen PM, Kemp SE, et al. ADAM17 in tumor associated leukocytes regulates inflammatory mediators and promotes mammary tumor formation. Genes Cancer. 2016;7(7-8):240-53.
33. Yang C, Zhao X, Cui N, Liang Y. Cadherins Associate with Distinct Stem Cell-Related Transcription Factors to Coordinate the Maintenance of Stemness in Triple-Negative Breast Cancer. Stem Cells Int. 2017;2017:5091541.
34. Chen C, Ridzon DA, Broomer AJ, Zhou Z, Lee DH, Nguyen JT, et al. Real-time quantification of microRNAs by stem-loop RT-PCR. Nucleic Acids Res. 2005;33(20):e179.
35. Cui J, Duan B, Zhao X, Chen Y, Sun S, Deng W, et al. MBD3 mediates epigenetic regulation on EPAS1 promoter in cancer. Tumour Biol. 2016;37(10):13455-67.
36. Dittmer J. The role of the transcription factor Ets1 in carcinoma. Semin Cancer Biol. 2015;35:20-38.
37. Thiagalingam A, De Bustros A, Borges M, Jasti R, Compton D, Diamond L, et al. RREB-1, a novel zinc finger protein, is involved in the differentiation response to Ras in human medullary thyroid carcinomas. Mol Cell Biol. 1996;16(10):5335-45.
38. Sanmartin E, Ortiz-Martinez F, Pomares-Navarro E, Garcia-Martinez A, Rodrigo-Banos M, Garcia-Escolano M, et al. CD44 induces FOXP3 expression and is related with favorable outcome in breast carcinoma. Virchows Arch. 2017;470(1):81-90.
39. Daum G, Eisenmann-Tappe I, Fries HW, Troppmair J, Rapp UR. The ins and outs of Raf kinases. Trends Biochem Sci. 1994;19(11):474-80.
40. Muller P, Crofts JD, Newman BS, Bridgewater LC, Lin CY, Gustafsson JA, et al. SOX9 mediates the retinoic acid-induced HES-1 gene expression in human breast cancer cells. Breast cancer research and treatment. 2010;120(2):317-26.
41. Moustakas A, Souchelnytskyi S, Heldin CH. Smad regulation in TGF-beta signal transduction. J Cell Sci. 2001;114(Pt 24):4359-69.
42. Gao N, Zhai Q, Li Y, Huang K, Bian D, Wang X, et al. Clinical Implications of TbetaRII Expression in Breast Cancer. PLoS One. 2015;10(11):e0141412.

43. Johnson E, Seachrist DD, DeLeon-Rodriguez CM, Lozada KL, Miedler J, Abdul-Karim FW, et al. HER2/ErbB2-induced breast cancer cell migration and invasion require p120 catenin activation of Rac1 and Cdc42. The Journal of biological chemistry. 2010;285(38):29491-501.
44. deGraffenried LA, Hilsenbeck SG, Fuqua SA. Sp1 is essential for estrogen receptor alpha gene transcription. J Steroid Biochem Mol Biol. 2002;82(1):7-18.
45. Garcia-Castro A, Zonca M, Florindo-Pinheiro D, Carvalho-Pinto CE, Cordero A, Gutierrez del Fernando B, et al. APRIL promotes breast tumor growth and metastasis and is associated with aggressive basal breast cancer. Carcinogenesis. 2015;36(5):574-84.
46. Nagasawa M, Gelfand EW, Lucas JJ. Accumulation of high levels of the p53 and p130 growth-suppressing proteins in cell lines stably over-expressing cyclin-dependent kinase 6 (cdk6). Oncogene. 2001;20(23):2889-99.
47. Cho YJ, Kang W, Kim SH, Sa JK, Kim N, Paddison PJ, et al. Involvement of DDX6 gene in radio- and chemoresistance in glioblastoma. International journal of oncology. 2016;48(3):1053-62.
48. Zeng S, Sun P, Chen Z, Mao J, Wang J, Wang B, et al. Association between single nucleotide polymorphisms in the ADD3 gene and susceptibility to biliary atresia. PLoS One. 2014;9(10):e107977.
49. Wu J, Zhang S, Shan J, Hu Z, Liu X, Chen L, et al. Elevated HMGA2 expression is associated with cancer aggressiveness and predicts poor outcome in breast cancer. Cancer Lett. 2016;376(2):284-92.
50. Zucchini C, Martinelli M, De Sanctis P, Rodia MT, Mattei G, Ugolini G, et al. Possible Gender-Related Modulation by the ROCK1 Gene in Colorectal Cancer Susceptibility. Pathobiology. 2015;82(6):252-8.
51. Lee M, Oprea-Ilies G, Saavedra HI. Silencing of E2F3 suppresses tumor growth of Her2+ breast cancer cells by restricting mitosis. Oncotarget. 2015;6(35):37316-34.
52. Cao Y, Zhou Z, de Crombrugghe B, Nakashima K, Guan H, Duan X, et al. Osterix, a transcription factor for osteoblast differentiation, mediates antitumor activity in murine osteosarcoma. Cancer Res. 2005;65(4):1124-8.
53. Deubzer HE, Schier MC, Oehme I, Lodrini M, Haendler B, Sommer A, et al. HDAC11 is a novel drug target in carcinomas. Int J Cancer. 2013;132(9):2200-8.
